# The *SYNGAP1* 3′UTR Variant in ALS Patients Causes Aberrant *SYNGAP1* Splicing and Dendritic Spine Loss by Recruiting HNRNPK

**DOI:** 10.1523/JNEUROSCI.0455-22.2022

**Published:** 2022-11-23

**Authors:** Satoshi Yokoi, Takuji Ito, Kentaro Sahashi, Masahiro Nakatochi, Ryoichi Nakamura, Genki Tohnai, Yusuke Fujioka, Shinsuke Ishigaki, Tsuyoshi Udagawa, Yuishin Izumi, Mitsuya Morita, Osamu Kano, Masaya Oda, Takefumi Sone, Hideyuki Okano, Naoki Atsuta, Masahisa Katsuno, Yohei Okada, Gen Sobue

**Affiliations:** ^1^Department of Neurology, Nagoya University Graduate School of Medicine, Nagoya 466-8550, Japan; ^2^Department of Neurology, Aichi Medical University School of Medicine, Aichi 480-1195, Japan; ^3^Department of Neural iPSC Research, Institute for Medical Science of Aging, Aichi Medical University, Aichi 480-1195, Japan; ^4^Public Health Informatics Unit, Department of Integrated Health Sciences, Nagoya University Graduate School of Medicine, Nagoya 461-8673, Japan; ^5^Division of ALS Research, Aichi Medical University, Aichi 480-1195, Japan; ^6^Research Division of Dementia and Neurodegenerative Disease, Nagoya University Graduate School of Medicine, Nagoya 466-8550, Japan; ^7^Graduate School of Pharmaceutical Sciences, Nagoya City University, Nagoya 467-8603, Japan; ^8^Department of Neurology, Institute of Biomedical Sciences, Tokushima University Graduate School, Tokushima 770-8503, Japan; ^9^Division of Neurology, Department of Internal Medicine, Jichi Medical University, Shimotsuke 329-0498, Japan; ^10^Department of Neurology, Toho University Faculty of Medicine, Tokyo 143-8540, Japan; ^11^Department of Neurology, Vihara Hananosato Hospital, Miyoshi 728-0001, Japan; ^12^Department of Physiology, Keio University School of Medicine, Tokyo 160-0016, Japan; ^13^Aichi Medical University, Aichi 480-1195, Japan

**Keywords:** amyotrophic lateral sclerosis, antisense oligonucleotides, dendritic spine, hnRNPK, iPSC-derived motor neuron, SYNGAP1

## Abstract

Fused in sarcoma (FUS) is a pathogenic RNA-binding protein in amyotrophic lateral sclerosis (ALS). We previously reported that FUS stabilizes Synaptic Ras-GTPase activating protein 1 (*Syngap1*) mRNA at its 3′ untranslated region (UTR) and maintains spine maturation. To elucidate the pathologic roles of this mechanism in ALS patients, we identified the *SYNGAP1* 3′UTR variant rs149438267 in seven (four males and three females) out of 807 ALS patients at the FUS binding site from a multicenter cohort in Japan. Human-induced pluripotent stem cell (hiPSC)-derived motor neurons with the *SYNGAP1* variant showed aberrant splicing, increased isoform α1 levels, and decreased isoform γ levels, which caused dendritic spine loss. Moreover, the *SYNGAP1* variant excessively recruited FUS and heterogeneous nuclear ribonucleoprotein K (HNRNPK), and antisense oligonucleotides (ASOs) blocking HNRNPK altered aberrant splicing and ameliorated dendritic spine loss. These data suggest that excessive recruitment of RNA-binding proteins, especially HNRNPK, as well as changes in *SYNGAP1* isoforms, are crucial for spine formation in motor neurons.

**SIGNIFICANCE STATEMENT** It is not yet known which RNAs cause the pathogenesis of amyotrophic lateral sclerosis (ALS). We previously reported that Fused in sarcoma (FUS), a pathogenic RNA-binding protein in ALS, stabilizes synaptic Ras-GTPase activating protein 1 (*Syngap1*) mRNA at its 3′ untranslated region (UTR) and maintains dendritic spine maturation. To elucidate whether this mechanism is crucial for ALS, we identified the *SYNGAP1* 3′UTR variant rs149438267 at the FUS binding site. Human-induced pluripotent stem cell (hiPSC)-derived motor neurons with the *SYNGAP1* variant showed aberrant splicing, which caused dendritic spine loss along with excessive recruitment of FUS and heterogeneous nuclear ribonucleoprotein K (HNRNPK). Our findings that dendritic spine loss is because of excess recruitment of RNA-binding proteins provide a basis for the future exploration of ALS-related RNA-binding proteins.

## Introduction

Fused in sarcoma (*FUS*) is one of the major causative genes for amyotrophic lateral sclerosis (ALS) and frontotemporal lobar degeneration (FTLD; Kwiatkowski et al., 2009; Neumann et al., 2009). The key pathologic findings of ALS-FUS or ALS/FTLD are the cytosolic mislocalization and aggregate formations of FUS (Neumann et al., 2009; Mackenzie et al., 2011), suggesting either the gain-of-toxic function (Sharma et al., 2016; López-Erauskin et al., 2018) or loss-of-function (Ishigaki and Sobue, 2018; Humphrey et al., 2020) of FUS. Abnormal liquid–liquid phase separation of FUS is also reported to be involved in the potential pathogenesis of FUS aggregation (Hofweber et al., 2018; Niaki et al., 2020).

FUS is an RNA-binding protein that is involved in various RNA metabolisms, including alternative splicing (Ishigaki et al., 2012; Lagier-Tourenne et al., 2012), mRNA stability (Kapeli et al., 2016), transcription (Tan and Manley, 2010; Masuda et al., 2015), mRNA transport (Fujii et al., 2005), and translation (Yasuda et al., 2013; Kamelgarn et al., 2018). FUS binds to the 3′ untranslated region (UTR) of its target mRNAs (Ishigaki et al., 2012; Lagier-Tourenne et al., 2012; Nakaya et al., 2013) and post-transcriptionally regulates mRNA expression (Udagawa et al., 2015; Yokoi et al., 2017; Akiyama et al., 2019; Garone et al., 2020). While aberrant RNA metabolism of FUS has been suggested to correlate with the pathogenesis of ALS/FTLD, it is still not clear which specific RNA pathway might directly cause ALS/FTLD.

Synaptic dysfunction is considered an initial pathologic change in ALS and various neurodegenerative diseases (Herms and Dorostkar, 2016; Henstridge et al., 2018). Importantly, FUS is known to be involved in synaptic function (Fujii et al., 2005; Qiu et al., 2014; Deshpande et al., 2019). We have investigated the relationship between FUS and synaptic function and found that FUS regulates the mRNA stability of Glutamate ionotropic receptor AMPA type subunit 1 (*Gria1*; Udagawa et al., 2015) and Synaptic Ras-GTPase activating protein 1 (*Syngap1*; Yokoi et al., 2017) at their 3′UTR, thus maintaining spine morphology and cognitive function. *SYNGAP1* is a pathogenic gene for intellectual disability including mental retardation, epilepsy, and autism spectrum disorders (Mignot et al., 2016), and SYNGAP1 is a major protein located at the postsynaptic density and negatively regulates the Ras/Rap pathway (Kim et al., 1998; Jeyabalan and Clement, 2016; Walkup et al., 2016). *Syngap1* knock-out mice exhibit an increased number of mature spines (Kim et al., 2003; Clement et al., 2012). SYNGAP1 has four isoforms that exert different synaptic functions and are defined by their C terminals: α1, α2, β, and γ (McMahon et al., 2012; Araki et al., 2020). For instance, the isoform α1 decreases synaptic strength, while α2 increases synaptic strength (McMahon et al., 2012). We previously reported that FUS specifically binds to the long 3′UTR of *Syngap1*, which is mainly connected to the *Syngap1* isoform α2 mRNA involved in strengthening synaptic function (Yokoi et al., 2017). Although these findings indicate that FUS-mediated *Syngap1* mRNA regulation is important for spine maturation in mice, its involvement in the pathogenesis of ALS/FTLD has never been described.

Therefore, we explored the whole exome sequencing data of the Japanese Consortium for Amyotrophic Lateral Sclerosis Research (JaCALS; a multicenter ALS cohort in Japan; Hayashi et al., 2020; Nakamura et al., 2021). We searched for a causative variant in the *SYNGAP1* 3′UTR binding site of FUS that might induce the aberrant binding properties of FUS, which cause aberrant *SYNGAP1* mRNA metabolism and synaptic dysfunction. As the *SYNGAP1* 3′UTR binding site of FUS in humans is different from that in mice (Nakaya et al., 2013), analyses using human-derived samples are mandatory for investigating the pathogenesis of the variant. Thus, the isogenic model of human-induced pluripotent stem cell (iPSC)-derived motor neurons were used to validate the pathogenic mechanism of the novel *SYNGAP1* variant in human ALS patients.

## Materials and Methods

### Study approval

The experiments using information and material related to patients with the *SYNGAP1* variant were approved by the ethics committee of Nagoya University Graduate School of Medicine (2004-0281). All the experimental procedures for the production and use of iPSCs were approved by the ethics committee of the Aichi Medical University School of Medicine (approval number 14-004, 2020-213).

### Genetic analysis

We performed whole exome sequencing in 807 patients with sporadic ALS and in 191 normal controls who had previously participated in the Japanese Consortium for Amyotrophic Lateral Sclerosis Research (JaCALS), which consists of 32 neurologic facilities. The details of JaCALS have been described elsewhere (Hayashi et al., 2020).

Genomic DNA was extracted from peripheral blood leukocytes. The exomes of patients with sporadic ALS were captured with the SureSelect Human All Exon V5+UTR or V6+UTR (Agilent Technologies). The libraries were indexed, pooled, and sequenced on an Illumina HiSeq 2000 Sequencer (paired-end, 100 base reads). The reads were aligned to a human reference genome (UCSC hg19) using BWA 0.6.2. The Picard tools 1.73 software was used to remove duplicate reads. Variants and insertions/deletions were identified with GATK 1.6–13 and filtered to the coordinates with variant quality score recalibration. NM_006772 (RefSeq) was referred to identify the 3′UTR region in the *SYNGAP1* gene, and variants within the FUS binding sites of the *SYNGAP1* 3′UTR were then selected. According to the CLIP-seq data of FUS in the human brain (Nakaya et al., 2013), the FUS binding sites were defined as those identified in multiple patients. The *SYNGAP1* 3′UTR variants (rs149438267) found in four males and three females were validated using Sanger sequencing. The sequence variants were validated by sequencing in both directions. There were no known pathogenic mutations for ALS in patients with the *SYNGAP1* 3′UTR variant. The sequencing details have been previously described (Nakamura et al., 2021).

### hiPSC culture and differentiation *in vitro*

The experiments were performed as described previously (Shimojo et al., 2015; Onodera et al., 2020; Okada et al., 2021). 201B7 cells (a gift from Shinya Yamanaka) were maintained on mitomycin-C-treated murin fibroblast STO cell line transformed with neomycin resistance and murine LIF genes (SNL) feeder cells in 0.1% gelatin-coated tissue culture dishes in human embryonic stem cell (hESC) medium, and were used to induce motor neurons. For differentiation, the SNL feeder cells were first removed. Then, hiPSC colonies were detached using a dissociation solution containing 0.25% trypsin, 100 μg/ml Collagenase IV, 1 mm CaCl_2_, and 20% knock-out serum replacement (KSR) medium, and were cultured in suspension in bacteriologic dishes in standard hESC medium without basic fibroblast growth factor for 1–2 h in gelatin-coated dishes. On day 1, the medium was changed to human embryoid body (hEB) medium containing DMEM/F-12, 5% KSR, 2 mm L-glutamine, 1% nonessential amino acids solution (NEAA) and 0.1 mm 2-mercaptoethanol with 300 nm LDN-193189, 3 μm SB 431542, and 3 μm CHIR 99 021. On day 2, the medium was changed to fresh hEB medium containing 300 nm LDN-193189, 3 μm SB431542, 3 μm CHIR99021, and 1 μm retinoic acid. On days 4–14, hEBs were cultured in hEB medium containing 1 μm retinoic acid and 1 μm purmorphamine; the medium was changed every 2–3 d. On day 14, hEBs were dissociated into single cells using TrypLE Select. The dissociated cells were plated on dishes coated with growth factor reduced Matrigel at a density of 1 × 10^5^ cells/cm^2^, and were cultured in motor neuron medium consisting of KBM Neural Stem Cell medium supplemented with 2% B27 supplement, 1% CultureOne supplement, 1% NEAA, 50 nm retinoic acid, 500 nm purmorphamine, 10 μm cyclic AMP, 10 ng/ml recombinant brain-derived neurotrophic factor (BDNF), 10 ng/ml recombinant glial cell line derived neurotrophic factor (GDNF), 10 ng/ml recombinant human insulin-like growth factor-1 (IGF-1), and 200 ng/ml L-ascorbic acid for up to four weeks. Half of the medium was changed every 2–3 d.

### Scarless genome editing in iPSCs

The donor plasmid (Yoshimatsu et al., 2019) was generated for scarless genome editing. The TTAA site in *SYNGAP1* 3′UTR was selected as the center of the 5′ and 3′ arm. The primers for cloning the *SYNGAP1* 3′UTR sequence are listed in Table 1. The plasmids pENTR2-L3-PBL-R1 (for the 5′ arm) and pENTR2-R2-PBR-L4 (for the 3′ arm) were linearized by HpaI and ligated with the cloned sequences by Gibson's assembly. To construct the donor plasmid, Multisite Gateway LR cloning was performed with the generated vectors with the arms as follows: pENTR-L1-PGK-PuroTK-L2 and pUC-DEST-R3R4. To generate single-guide RNAs (sgRNAs) targeting the *SYNGAP1* variant, the Guide-it sgRNA In Vitro Transcription kit (Takara) was used according to the manufacturer's instructions. We generated sgRNA targeting AAGAGAGGGCAGCACCCCAATGG to generate the homozygous mutation and CCCCCTTTTCCTTCCCATTGGGG to generate the heterozygous mutation. Y-27 632 (10 μm) and valproic acid (10 μm) were added to iPSC culture medium 24 h before electroporation. On day 1, iPSCs were dissociated from SNL feeder cells into single cells using TrypLE Select (Thermo Fisher Scientific). Guide-it Recombinant Cas9 (Takara) was used for the Cas9-sgRNA ribonucleoprotein (RNP) complex. RNP, donor plasmid, and Rad51-expressing plasmid (a kind gift from Mizuguchi; Takayama et al., 2017) were introduced into iPSCs by electroporation according to the manufacturer's instructions. The iPSCs were then plated onto three 10 cm dishes in twofold dilution. On days 3 and 4, puromycin (10 μm) was added for the selection process. After two weeks, single colonies were picked, and the *piggyBac* cassette insertion was screened by Sanger sequences. For *piggyBac* transposition, the pCAGS-PBx plasmid was introduced into the cell lines with optimal *piggyBac* cassette insertion by electroporation. Next, ganciclovir (2.5 μg/ml) was added on days 2–4 after electroporation. After two weeks, single colonies were picked and screened by Sanger sequences and restriction enzyme treatment (Msl1). The karyotypes were confirmed to be normal in all the edited cell lines (data not shown).

**Table 1.** List of primers, oligonucleotides, and antibodies used in this study

Primers		Source
3′RACE F primer GATTACGCCAAGCTTGCGGAGGCTGCTGTCCCAGGAAGAACAA		This study
3′UTR DNA F primer: TCTGGTTTCCTGGTGTGACA		This study
3′UTR DNA R primer: AACGCAAAAGGATGAAGGGG		This study
3′UTR F primer: TGGAAGAGTATGAGCGGAGG		This study
3′UTR R primer: TTCACCACACACTCCAGAAAA		This study
*SYNGAP1* ORF F primer: ATCGATGGATGAGAGCCGG		This study
*SYNGAP1* ORF R primer: TTTGCTGGTTTGTTCTTCCTGG		This study
Fragment analysis F primer: TGGAGCAGAGTGAGAAGAGGC		This study
Fragment analysis R primer: GTCACACGCGGGTTTGTTG		This study
*FUS* F primer: GTGGAGGCTATGGAGGAGGT		This study
*FUS* R primer: GCCTTACACTGGTTGCATTC		This study
*HNRNPK* F primer: TGCTGTCCTCATTCCACTGAC		This study
*HNRNPK* R primer: TTGGGATCATAAGGCTGTGCA		This study
*GAPDH* F primer: ACAGTCAGCCGCATCTTCTT		This study
*GAPDH* R primer: ACGACCAAATCCGTTGACTC		This study
β*-Actin* F primer: CTCTTCCAGCCTTCCTTCCT		This study
β*-Actin* R pirmer: TGTTGGCGTACAGGTCTTTG		This study
5′arm F primer for cloning *SYNGAP1* 3′UTR CAACTTTGTATAATAAAGTTGGAGAGGCAGCTTCCCCCCTTG		This study
5′arm R primer for cloning *SYNGAP1* 3′UTR ATGATTATCTTTCTAGGGTTAAGAGTGGTAGAAGGAGAAGGA		This study
3′arm F primer for cloning *SYNGAP1* 3′UTR CAGACTATCTTTCTAGGGTTAAATTTACTCCCTCCCCACCCA		This study
3′arm R primer for cloning *SYNGAP1* 3′UTR CAACTTTGTATAGAAAAGTTGGAGCAACTCCCTACCCTTGAGG		This study
Oligonucleotides		Source
SYNGAP1-ASO: CUGGGGGUCAAAGAGAGGGC		This study
Ctrl-ASO: AUGAGUGCCAGAGCGAUGUC		This study
E1-ASO: GGTCCCUGGGGGUCAAAGAG		This study
E3-ASO: CAAGGGUCCCUGGGGGUCAA		This study
shFUS #1: GGACAGCAGCAAAGCTATA		This study
shFUS #2: CGGACATGGCCTCAAACGA		This study
shHNRNPK #1: GCATTTAAAAGATCTAGAA		This study
shHNRNPK #2: GGTTTCAGTGCTGATGAAA		Liu et al. (2018)
shCtrl: AATTCTCCGAACGTGTCACGT		This study
Antibodies	Source	Identifier
Rabbit polyclonal anti-FUS antibody	Bethyl Laboratories	Catalog #A300-293A, RRID:AB_263409
Mouse monoclonal anti-FUS antibody	Santa Cruz Biotechnology	Catalog #sc-47711, RRID:AB_2105208
Rabbit polyclonal anti-PAN SYNGAP1	Thermo Fisher Scientific	Catalog #PA1-046, RRID:AB_2287112
Rabbit polyclonal anti-SYNGAP1 α1	Millipore	Catalog # 06–900, RRID:AB_11212750
Rabbit polyclonal anti-SYNGAP1 α2	Abcam	Catalog #ab77235, RRID:AB_1524465
Rabbit polyclonal anti-HNRNPK	MBL international	Catalog #RN019P, RRID:AB_1953048
Mouse monoclonal anti-HNRNPK	Santa Cruz Biotechnology	Catalog #sc-28380, RRID:AB_62773
Chicken polyclonal anti-CHAT	Aves Labs	Catalog #CAT, RRID:AB_2313537
Mouse monoclonal anti-HB9	DSHB	Catalog #MNR2, RRID:AB_2314625
Mouse monoclonal anti-ISL1	DSHB	Catalog #PCRP-ISL1-1D4, RRID:AB_2618777
Rabbit polyclonal anti-βIII-Tubulin	BioLegend	Catalog #845502, RRID:AB_2566589
Chicken polyclonal anti-MAP2	Abcam	Catalog #ab5392, RRID:AB_2138153
Mouse monoclonal anti-GAPDH	MBL International	Catalog #M171-3, RRID:AB_10597731
Rabbit polyclonal anti-ELAVL2	Proteintech	catalog # 14008–1-AP, RRID:AB_2096356
Mouse monoclonal anti-PUF60	GeneTex	Catalog #GTX629887, RRID:AB_2888176
Rabbit polyclonal anti-synaptophysin	Abcam	Catalog #ab32127, RRID:AB_2286949
Alexa Fluor 488 phalloidin	Thermo Fisher Scientific	N/A
Alexa Fluor 555 anti-chicken secondary antibody	Thermo Fisher Scientific	Catalog #A-21437, RRID:AB_2535858
Alexa Fluor 633 anti-rabbit secondary antibody	Thermo Fisher Scientific	Catalog #A-21070, RRID:AB_2535731
Alexa Fluor 633 anti-chicken secondary antibody	Thermo Fisher Scientific	Catalog #A-21103, RRID:AB_2535756
Alexa Fluor 488 anti-mouse IgG2a secondary antibody	Thermo Fisher Scientific	Catalog #A-21131, RRID:AB_2535771
Alexa Fluor 555 anti-mouse IgG1 sary antibody	Thermo Fisher Scientific	Catalog #A-21127, RRID:AB_2535769

### Antisense oligonucleotide (ASO) treatment

For this treatment, 2′-O-methyl RNA/DNA oligonucleotides with a phosphorothioate backbone were generated by Integrate DNA Technologies (IDT). Two thirds of the nucleotides were modified in 2′-O-methyl RNA. Next, the ASOs (50 nm) were added directly to the culture medium without any transfection reagents on day 14 after plating the motor neuron cultures.

### Biotinylated RNA pull-down assay

To assess the interaction of *SYNGAP1* mRNA 3′UTR with endogenous RNA-binding proteins, a biotinylated RNA pull-down assay was performed as described in previous studies (Udagawa et al., 2015; Yokoi et al., 2017). The *SYNGAP1* 3′UTR sequence was amplified from the plasmids produced in the 3′ rapid amplification of cDNA ends (RACE) of wild-type (WT) iPSC-derived motor neurons. The mutation was introduced using the PrimeSTAR Mutagenesis Basal kit (Takara), according to the manufacturer's instructions. DNA templates were amplified using RT-PCR and the primers 5′-TAATACGACTCACTATAGGGCCCACCCAGCATCAGAGACC-3′ and 5′-GTCCCTGGGGGTCAAAGAGA-3′, and were purified using a PCR purification kit (QIAGEN) as a template for *in vitro* transcription. T7 RNA polymerase (Takara) with biotin RNA labeling mix (Roche) was used for *in vitro* transcription according to the manufacturer's instructions. The lysate containing 300 μg of the proteins from the motor neuron cultures was incubated with 3 μg biotinylated RNA for 1 h at room temperature; then, streptavidin Dynabeads (Invitrogen) were added. After 1 h of incubation at 4°C, the beads were washed with Brain IP buffer three times, boiled with 4× NuPAGE LDS-PAGE sample buffer (Novex) containing β-mercaptoethanol for 5 min at 95°C, and analyzed with Western blotting. The band intensities of the input and pull-down were quantified.

### Liquid chromatography-mass spectrometry (LC-MS/MS) analysis

For the LC-MS/MS analysis, proteins from the pull-down assay were stained with the Silver Stain kit (Invitrogen), and the bands of interest were excised and destained according to the manufacturer's instructions. After reduction and alkylation, the proteins were digested by trypsin for 16 h at 37°C. The peptides were analyzed using LC-MS with an Orbitrap Fusion mass spectrometer (Thermo Fisher Scientific) coupled to an UltiMate3000 RSLCnano LC system (Dionex Co) using a nano HPLC capillary column [150 mm × 75 μm inner diameter (i.d.); Nikkyo Technos Co] via a nanoelectrospray ion source. Reversed-phase chromatography was performed with a linear gradient (0 min, 5% B; 100 min, 40% B) of solvent A (2% acetonitrile with 0.1% formic acid) and solvent B (95% acetonitrile with 0.1% formic acid) at an estimated flow rate of 300 nl/min. A precursor ion scan was conducted using a 400–1600 mass-to-charge ratio (m/z) before MS/MS analysis. Tandem MS was performed by isolation at 0.8 Th with the quadrupole, HCD fragmentation with a normalized collision energy of 30%, and rapid scan MS analysis in the ion trap. Only those precursors with a charge state of 2–6 were sampled for MS2. The dynamic exclusion duration was set to 15 s with a tolerance of 10 ppm. The instrument was run in top-speed mode with 3-s cycles. The raw data were processed using Proteome Discoverer 1.4 (Thermo Fisher Scientific) in conjunction with the MASCOT search engine, version 2.7.0 (Matrix Science), for protein identification. Peptides and proteins were identified against the human protein database in UniProt (release 2021_01), with a precursor mass tolerance of 10 ppm and a fragment ion mass tolerance of 0.8 Da. The fixed modification was set to cysteine carbamidomethylation, and the variable modifications were set to methionine oxidation. Two missed cleavages by trypsin were allowed. LC/MS/MS data of pull-down assay in Extended Data Table 4-1 have been deposited at PRIDE and are publicly available as of the date of publication (project accession: PXD026586; project DOI: 10.6019/PXD026586).

### Quantitative analysis of immunofluorescence data

The immunofluorescent signal intensity was measured by ZEN software (Zeiss). The quantification of spine number was performed as described previously (Yokoi et al., 2017). Regions of dendrites located 30–100 µm from the cell soma that did not intersect with other dendrites were selected. The numbers of spines, labeled with Alexa Fluor 488-conjugated phalloidin, were counted over a region of 20 µm in length. The number of spines was counted independently, without sample information. Synaptophysin-positive F-actin particles were detected using Zen software. The differentiation efficacy of iPSC-derived motor neurons was calculated by ImageJ. The intensities were calculated, and cells with intensities above the same threshold were considered positive.

### 3′RACE and TA-cloning

To identify the *SYNGAP1* 3′UTR variants, we used the SMARTer RACE 5′/3′ kit (Takara) according to the manufacturer's instructions. The primer used in the assay was 5′-CTGTCCCAGGAAGAACAAACCAGCAA-3′. The PCR products were inserted into the pRACE vector within the kit, and the sequences were analyzed.

### Immunocytochemistry and F-actin staining of neuronal cultures

Cultured motor neurons were immunostained as described previously (Yokoi et al., 2017), with some modifications. Samples were fixed with 4% paraformaldehyde at 37°C for 10 min, washed with PBS twice, and blocked with 50% normal goat serum and 0.2% Triton X-100 (in PBS) for 30 min. Primary antibodies were diluted in 10% normal goat serum and 0.1% Triton X-100 (in PBS). After overnight incubation at 4°C with the primary antibodies, neurons were washed three times with PBS, and incubated with Alexa Fluor 488-, 555-, and 633-conjugated secondary antibodies (Invitrogen) at a dilution of 1:1000 for 2 h at room temperature. To evaluate spine morphology, F-actin was stained by Alexa Fluor 488-conjugated phalloidin (1:200, Invitrogen). Images were acquired with a microscope (LSM 710, Zeiss) at 40× or 100× magnification with oil immersion.

### Western blotting

Samples were homogenized with brain immunoprecipitation buffer containing 25 mm HEPES-NaOH (pH 7.4), 150 mm NaCl, 5 mm MgCl_2_, 1 mm EDTA, and 1% NP-40. Protease inhibitors (Roche), phosphatase inhibitors (Thermo Fisher Scientific), and RNase inhibitors (Takara) were supplemented when needed. Homogenates were incubated on ice for 20 min and centrifuged at 13,000 rpm for 10 min at 4°C. The lysates were mixed with 4× NuPAGE lithium dodecyl sulfate (LDS) sample buffer (Novex), heated at 70°C for 5 min, and analyzed by Western blotting using the antibodies listed in Table 1. The band intensities were quantified using Multi Gauge 3.0 software (FujiFilm).

### Immunoprecipitation

The total cell lysates were mixed with the antibodies and rotated at 4°C for 30 min. Protein-G Dynabeads (Invitrogen) were added to the mixture and further incubated at 4°C for 1.5 h. After washing the beads with Brain immunoprecipitation buffer three times, bound proteins or RNA were analyzed as below. The beads were directly mixed with NuPAGE LDS-PAGE sample buffer containing β-mercaptoethanol, heated at 95°C for 5 min, and analyzed with Western blotting. To analyze the bound RNA, the buffer was supplemented with RNase inhibitor (Takara). The beads after immunoprecipitation were incubated with 100-µl protein K buffer (100 mm Tris-HCl, pH 7.5, 12.5 mm EDTA, 150 mm NaCl, 1% SDS, 2 μg/μl protein K) for 15 min at 65°C. Then, Tris-EDTA buffer (200 µl) and phenol (300 µl) were added and vortexed vigorously. The RNA collected in the aqueous fraction was chloroform-washed and pelleted using ethanol precipitation with glycogen. The RNA was resuspended in distilled water and analyzed using quantitative RT-PCR.

### Quantitative real-time PCR

RNA was prepared from motor neuron cultures using the Rneasy Mini kit (QIAGEN), according to the manufacturer's instructions. Total RNA was used as the template for reverse transcription using Superscript IV (Invitrogen). qRT-PCR was performed using the KAPA SYBR FAST qPCR kit (Kapa Biosystems), according to the manufacturer's instructions. RNA levels were normalized to those of β*-actin* mRNA.

### DNA constructions

Targeting sequences of shRNA were listed in Table 1. These were cloned into a lentiviral shRNA vector (pLenti-RNAi-X2 puro DEST). For lentiviral SYNGAP1 α1 and γ expression, the plasmid containing the human SYNGAP1 α2 cDNA sequence (Dana form) was modified and cloned into the pLenti CMV-TO DEST puro vector with the FLAG tag. The sequences of shRNA were listed in Table 1. The sequence of shHNRNPK2 was adapted from that previously reported (Liu et al., 2018).

### Lentivirus production

Lentiviruses were produced as reported previously (Yokoi et al., 2017). The lentiviral transfer vector mentioned above with the packaging vectors pLP1, pLP2, and pVSVG was transfected into HEK293T cells. Lipofectamine 2000 in Opti-MEM was used for transfection according to the manufacturer's instructions. The medium was replaced with fresh Opti-MEM 1 d after transfection and collected with filtration 1 d later.

### Statistical analyses

Western blotting, immunocytochemistry, immunoprecipitation, and qRT-PCR data were obtained from at least three independent experiments. All data were analyzed using IBM SPSS Statistics 27. Normality was determined using the Shapiro–Wilk test. For the statistical analysis of two groups, the unpaired *t* test was used. When the data were not distributed normally, the Mann–Whitney *U* test was used. In experiments with more than two groups, one-way analysis of variance (ANOVA) with the *post hoc* Tukey's multiple comparison test was used. When the data were not distributed normally, the Kruskal–Wallis test and *post hoc* Bonferroni test were used; χ^2^ test and Fisher's exact test were used to test the independence of frequencies. In all experiments, the data are expressed as mean ± standard error of the mean (SEM), and the thresholds of statistical significance were set to *p* < 0.05, *p* < 0.01, and *p* < 0.001.

## Results

### The *SYNGAP1* 3′UTR variant at the FUS binding site was identified in the Japanese ALS cohort

**Figure 1.**
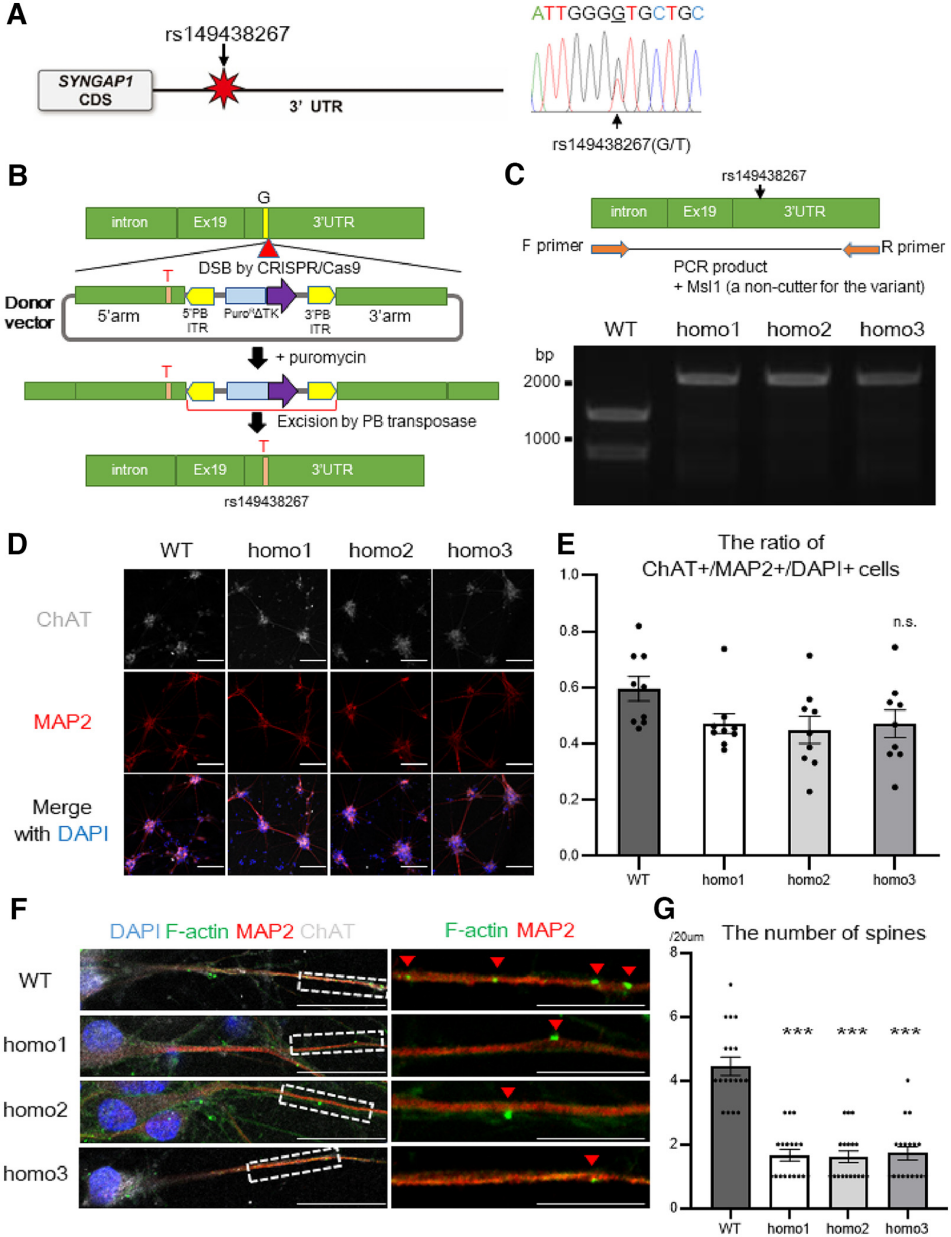
Motor neurons with the *SYNGAP1* rs149438267 homozygous mutation exhibit a loss of dendritic spines. ***A***, The location of the *SYNGAP1* variant rs149438267 (g.33452118G>T, c.*212G>T, GRCh38.p12; left). Sanger sequence of a patient with the *SYNGAP1* 3′UTR variant rs149438267 (right). Genomic DNA were extracted from peripheral blood leukocytes, and the heterozygous variant g.33452118G>T was confirmed. ***B***, A schematic overview showing the strategy for scarless genome editing, inserting rs149438267 into iPSCs (201B7). ***C***, RT-PCR was performed using primer sets to amplify DNA extracted from wild-type and edited homozygous iPSCs. PCR products were digested by Msl1, a noncutter for the *SYNGAP1* mutation, and analyzed by agarose gel electrophoresis. ***D***, iPSC-derived motor neurons with the wild-type or homozygous mutation were cultured for four weeks and were immunostained for MAP2 (red), ChAT (white), and DAPI (blue). Scale bar: 10 µm. ***E***, Quantification of the ratio of ChAT/MAP2/DAPI-positive cells. Data are presented as the mean ± SEM *n* = 9 fields, each from 3 independent wells; n.s., not significant, Kruskal–Wallis test, Bonferroni *post hoc* test. ***F***, iPSC-derived motor neurons were immunostained for F-actin (green), MAP2 (red), and ChAT (white). Scale bars: 20 µm for left columns, 10 µm for right columns. ***G***, Quantification of the number of spines per 20 µm of dendrite length. Data are presented as the mean ± SEM *n* = 18 each; ***p < 0.001, one-way ANOVA, Tukey's *post hoc* test. Additional data of the FUS binding sites at *SYNGAP1* 3′UTR and induced pluripotent stem cell (iPSC)-derived motor neurons are displayed in Extended Data Figure 1-1.

First, we referred to the CLIP-seq data of FUS in the human brain (Nakaya et al., 2013) for the FUS binding sites of human *SYNGAP1* 3′UTR. We found that most of the FUS CLIP tags were located in relative close proximity to exon 19, the 5′ side of 3′UTR (Extended Data Fig. 1-1*A*), unlike the FUS CLIP tags in mice that are mostly located at the distant side to the exons at the 3′ side of 3′UTR (Nakaya et al., 2013; Yokoi et al., 2017). This indicates that FUS regulation of *SYNGAP1* mRNA might differ between humans and mice. Based on these findings, we examined the *SYNGAP1* 3′UTR in the whole exome sequencing data of 807 Japanese patients with sporadic ALS and 191 normal controls registered in JaCALS database. We found eight variants in the *SYNGAP1* 3′UTR and the heterozygous *SYNGAP1* 3′UTR variant rs149438267 (G to T) in seven out of 807 ALS patients (1614 alleles), while it was absent in all 191 controls (382 alleles; Fig. 1*A*; Table 2), was the only variant at the FUS binding site. The allele frequency of this variant was 0.00,434, which was significantly higher than those in the nation-wide Japanese database ToMMO8.3KJPN (0.0017, *p* = 0.037, Fisher's exact test) and GEMJ-WGA (0.0017, *p* = 0.036, Fisher's exact test; Tadaka et al., 2018; Table 2). Interestingly, the frequency of the variant was higher in the Japanese database compared with TOPMED or gnomAD (Karczewski et al., 2020), suggesting different incidence rates among different populations. In ALS patients with this variant, upper limb weakness or dysarthria were frequently observed as initial symptoms, whereas cognitive functions were well preserved (Table 3). The clinical courses were relatively rapid, especially in patients with dysarthria during the initial stage of the disease.

**Table 2.** The allele frequency of the *SYNGAP1* variant rs149438267

Population	Allele count	Frequency of G/T allele^*a*^
JaCALS patient group	1614	0.00434*
JaCALS control group	382	0
ToMMo8.3KJPN	16,760	0.0017
GEM-JWGA^*b*^	14,452	0.0017
ToPMeD (phase 3)	125,568	0.00014
gnomAD (v2.1.1)	31,136	0.000032
European (non-Finnish)	15,280	0.000065
African	8634	0
Latino	846	0
Ashkenazi Jewish	290	0
East Asian	1552	0
European (Finnish)	3458	0
Other	1076	0

*p < 0.05 (*p* = 0.037, compared with ToMMo 8.3KJPN; *p* = 0.036, compared with GEM-J WGA, Fisher's exact test).

*^a^*All alleles are heterozygotes.

*^b^*The filter status of GEM-J WGA is not high confident region.

**Table 3.** The clinical features of ALS patients with *SYNGAP1* variant rs149438267

Sex	M	F	M	F	M	M	F
Age at onset	64	57	66	75	66	77	75
Clinical diagnosis based on the revised El Escorial criteria	Probable laboratory supported	Probable laboratory supported	Definite	Probable	Probable	Definite	Probable
Initial symptoms	Upper limb weakness	Upper limb weakness	Dysarthria	Upper limb weakness	Dysarthria, dysphagia, dyspnea	Dysarthria	Dysarthria
Duration from onset to death (month)	30	64	9	29 (censored observation)	7	18	38
Upper motor symptom	+	+	+	+	+	+	+
Lower motor symptom	+	+	+	+	+	+	+
Active denervation in EMG	+	+	+	+	+	+	+
Chronic denervation in EMG	+	+	+	+	+	+	−
Dementia	−	+	−	−	−	−	−
Cognitive tests	Not examined	MMSE 23, FAB 7	Not examined	MMSE 26, FAB 13	Not examined	MMSE 20, FAB 14	Not examined

### The *SYNGAP1* homozygous mutation induced a loss of dendritic spines

To reproduce the relationship between RNA-binding proteins and *SYNGAP1* 3′UTR by a single-nucleotide polymorphism (SNP) of noncoding sequence, we performed scarless genome editing by CRISPR-Cas9 and inserted the *SYNGAP1* 3′UTR variant rs149438267 into iPSCs in isogenic condition (Fig. 1*B*). We chose 201B7 because they have been well characterized (Takahashi et al., 2007) and because they are one of the most commonly and widely used high quality iPSCs (Imamura et al., 2017; Fujimori et al., 2018; Akiyama et al., 2019). First, we generated iPSCs carrying the rs149438267 variant in homozygosis to analyze the full genetic effect expressed by this polymorphism (Fig. 1*C*; Extended Data Fig. 1-1*B*). These screened iPSCs were then differentiated into motor neurons (Extended Data Fig. 1-1*C*; Shimojo et al., 2015; Onodera et al., 2020; Okada et al., 2021), and cell differentiation efficiencies were evaluated using the following procedure: after one week by immunocytochemistry of Islet-1 (ISL-1), homeobox protein HB9 (HB9), and β-III-tubulin (Extended Data Fig. 1-1*D*,*E*), and after four weeks by choline acetyltransferase (ChAT) and microtubule-associated protein 2 (MAP2; Fig. 1*D*,*E*). These data suggested that the differentiation efficiencies were similar in the wild-type (WT) and the homozygous mutant iPSC-derived motor neurons. Next, we evaluated spines in the motor neurons using F-actin immunolabeling (Extended Data Fig. 1-1*F*,*G*), and the number of F-actin particles was significantly decreased in the rs149438267 homozygous motor neurons (Fig. 1*F*,*G*). These results indicate that the *SYNGAP1* 3′UTR variant rs149438267 from patients with ALS is involved in the spine formation of motor neurons.

### The *SYNGAP1* homozygous mutant increased isoform α1 levels without altering total expression of SYNGAP1

**Figure 2.**
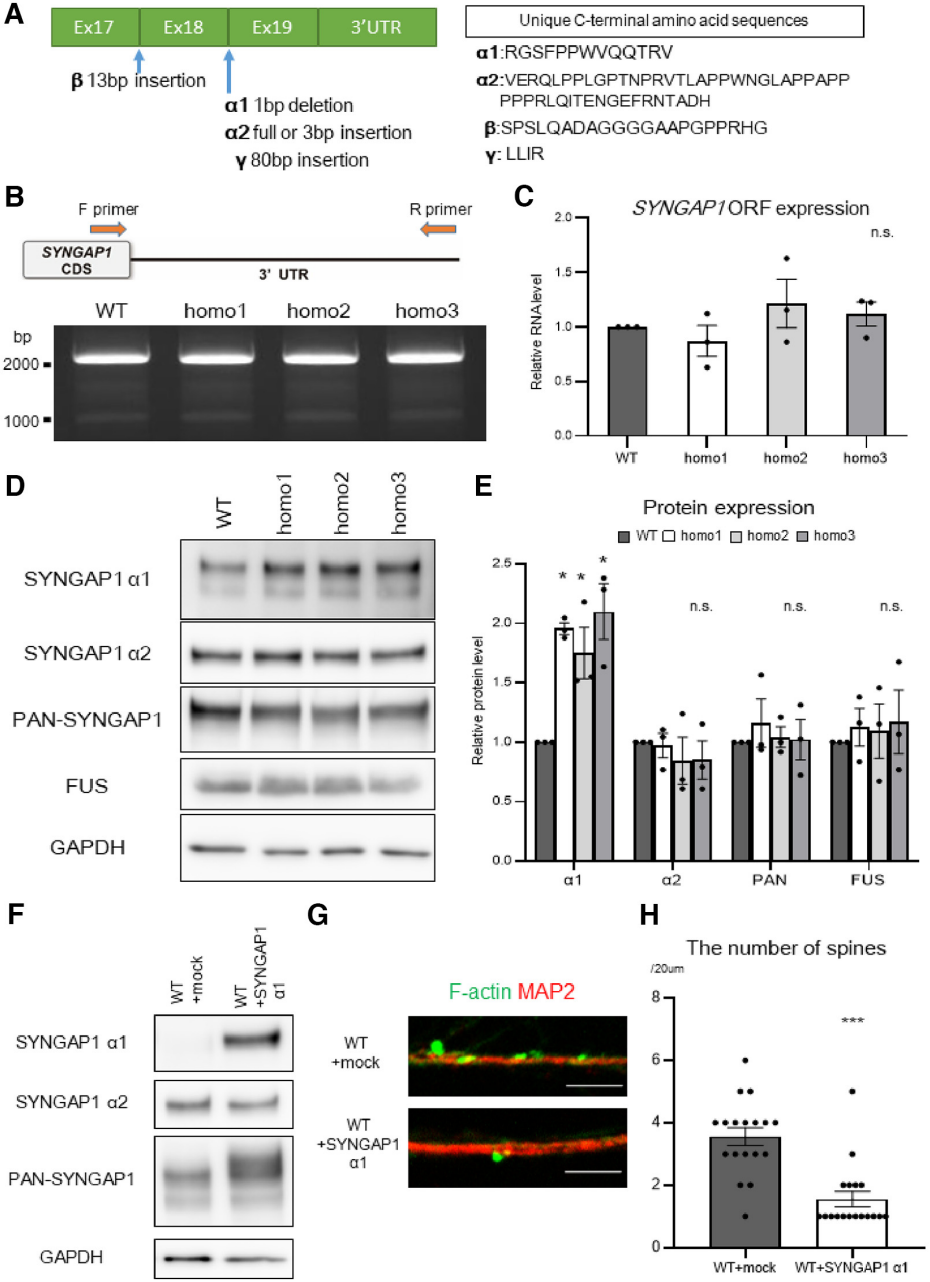
The *SYNGAP1* mutation increases isoform α1 protein levels, which decreases the number of spines. ***A***, A schematic overview of the spliced variant of *SYNGAP1* with C-terminal exons (green columns). ***B***, RNA from wild-type and homozygous motor neurons was analyzed using RT-PCR to evaluate 3′UTR splicing. PCR products were analyzed with agarose electrophoresis to evaluate the 3′UTR variants. Representative data from triplicate experiments are shown. Additional data of *SYNGAP1* 3′UTR in iPSC-derived motor neurons are displayed in Extended Data Figure 2-1. ***C***, RNA from wild-type and homozygous motor neurons was analyzed with qRT-PCR and the primer set for *SYNGAP1*-ORF. Data are presented as the mean ± SEM *n* = 3 each; n.s., not significant, one-way ANOVA, Tukey's *post hoc* test. ***D***, The lysates from wild-type and homozygous iPSC-derived motor neurons were analyzed by Western blotting using the indicated antibodies. ***E***, Quantification of the band intensities of the proteins in ***D***. *n* = 3; *p < 0.05; n.s., not significant, one-way ANOVA, Tukey's *post hoc* test. ***F***, Wild-type iPSC-derived motor neurons were infected with lentiviruses containing *SYNGAP1* isoform α1 expression vector or mock vector. The lysates were analyzed by Western blotting using the indicated antibodies. Representative data from triplicate experiments are shown. ***G***, Wild-type iPSC-derived motor neurons were infected with lentiviruses containing *SYNGAP1* α1 expression vector or mock vector, and were immunostained for F-actin (green) and MAP2 (red). Scale bar: 5 µm. ***H***, Quantification of the number of spines per 20 µm. Data are presented as the mean ± SEM *n* = 18 each; ***p < 0.001, Mann–Whitney *U* test.

SYNGAP1 has four isoforms (α1, α2, β, and γ), based on its spliced variant between exon 17, 18, and 19 (Fig. 2*A*; Jeyabalan and Clement, 2016; Kilinc et al., 2018). Each splice variant results in frameshifts and coded specific C-terminal amino acid sequences. Moreover, 3′UTR connected to each open reading frame (ORF) sequence is also known to have spliced variant in mice (Yokoi et al., 2017). First, we analyzed the spliced variants of *SYNGAP1* 3′UTR using rapid amplification of cDNA ends (RACE) of the RNA sequences from the WT iPSC-derived motor neurons. We found that short 3′UTR was present but to a lesser extent than full-length 3′UTR (Extended Data Fig. 2-1*A*). Cloning of RACE fragments revealed that the major ORF isoform of human *SYNGAP1* was α2, coinciding with that of mice (Yokoi et al., 2017), and that the ORF isoform ratio between full-length 3′UTR and spliced short UTR was similar (Extended Data Fig. 2-1*B*). Unexpectedly, short UTR has many variations of potential spliced isoforms (Extended Data Fig. 2-1*C*). According to these data, we performed RT-PCR from *SYNGAP1*-ORF to the end of 3′UTR, and found no differences in the proportion of each isoform between the WT and the rs149438267 homozygous motor neurons (Fig. 2*B*). Although the *SYNGAP1* variant rs149438267 was located at the 3′UTR, the results suggest that this variant did not affect the splicing pattern of *SYNGAP1* 3′UTR.

Next, we focused on ORF expression of *SYNGAP1*. qRT-PCR of *SYNGAP1*-ORF revealed that ORF expression levels remained unchanged in the rs149438267 homozygous motor neurons (Fig. 2*C*). Western blotting showed an increase in SYNGAP1 isoform α1, while PAN-SYNGAP1 and SYNGAP1 α2 remained unchanged (Fig. 2*D*,*E*), suggesting that the increase in SYNGAP1 isoform α1 might be because of changes in alternative splicing. Note that the only commercially available antibodies are against PAN-SYNGAP1 and α1 and α2 isoforms. We also generated iPSCs with the heterozygous mutation (Extended Data Fig. 2-1*D*) but could not detect the same alteration in SYNGAP1 isoform expression (Extended Data Fig. 2-1*E*,*F*). This suggests that the heterozygous mutation might exert less effect on the SYNGAP1 isoform under the current culture conditions.

SYNGAP1 isoform α1 is reported to decrease synaptic strength (Rumbaugh et al., 2006; McMahon et al., 2012), and we also reported that SYNGAP1 α1 overexpression decreases the number of mature spines in mouse primary hippocampal neurons (Yokoi et al., 2017). To confirm the effect of SYNGAP1 α1 on synaptic function, we overexpressed SYNGAP1 α1 in the WT motor neurons (Fig. 2*F*) and confirmed that SYNGAP1 α1 decreased the number of spines (Fig. 2*G*,*H*). These results suggest that an increase in SYNGAP1 α1 expression causes dendritic spine loss in the rs149438267 homozygous motor neurons.

### The *SYNGAP1* variant decreased SYNGAP1 isoform γ, which exerted a positive effect on spine formation

**Figure 3.**
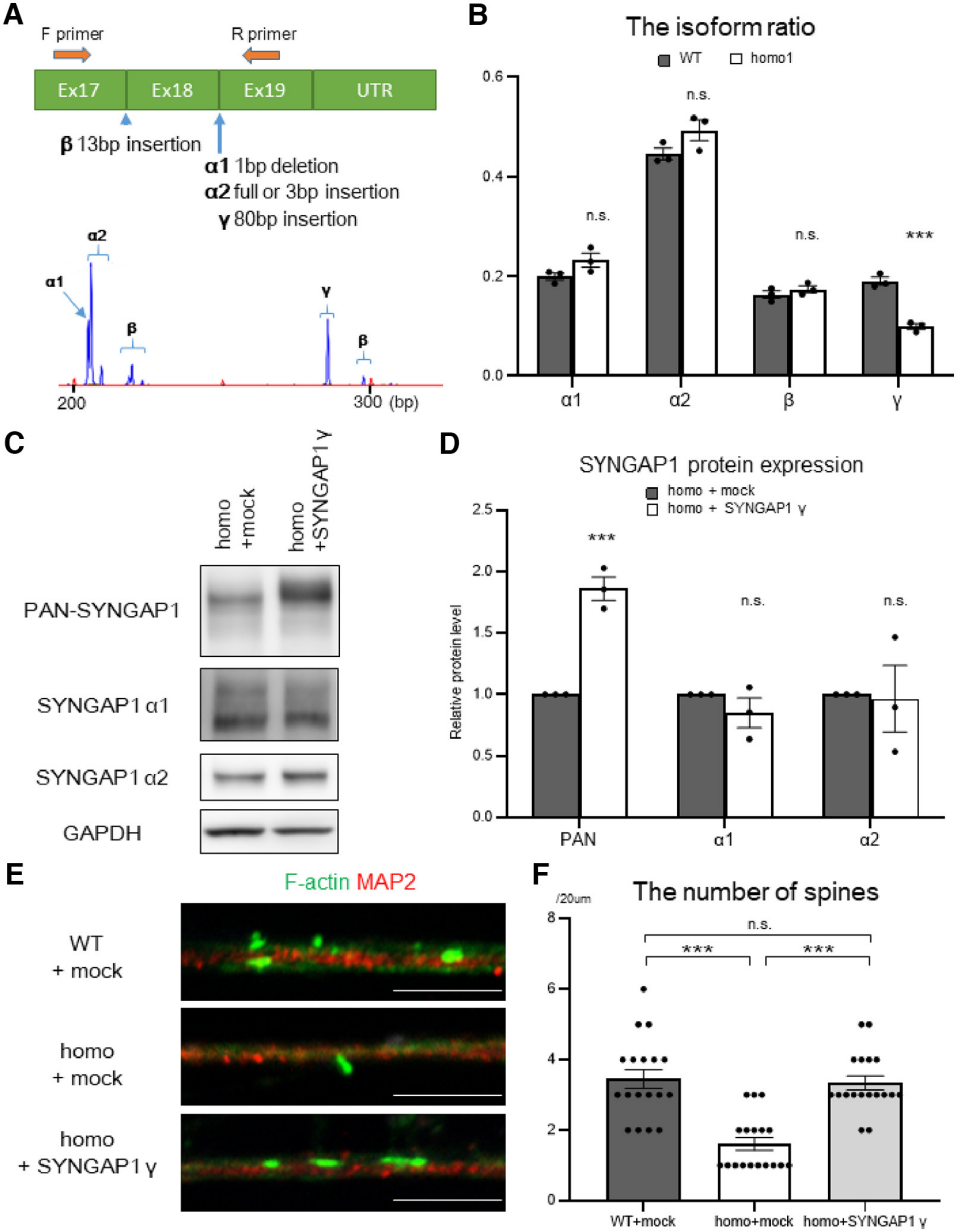
The *SYNGAP1* mutation decreases *SYNGAP1* isoform γ and thereby depletes the number of spines. ***A***, A schematic overview of fragment analysis. The primer set was placed between exon 17 and exon 19 to evaluate spliced variants simultaneously. Representative raw data of fragment analysis are shown. ***B***, RNA from wild-type or homozygous 1 (homo1; hereafter homo) motor neurons was analyzed with RT-PCR, and the PCR products were analyzed with fragment analysis. Data are presented as the mean ± SEM *n* = 3 each; ***p < 0.001; n.s., not significant, unpaired *t* test. Additional data of the isoform changes in motor neurons with *SYNGAP1* homozygous mutation are displayed in Extended Data Figure 3-1. ***C***, iPSC-derived motor neurons with the homozygous mutation were infected with lentiviruses expressing *SYNGAP1* isoform γ or mock. The lysates were analyzed with Western blotting using the indicated antibodies. ***D***, Quantification of the band intensities of the proteins in ***C***. Data are presented as the mean ± SEM *n* = 3; ***p < 0.001; n.s., not significant, unpaired *t* test. ***E***, Wild-type or homozygous iPSC-derived motor neurons were infected with lentiviruses expressing SYNGAP1 isoform γ or mock, and were immunostained for F-actin (green) and MAP2 (red). Scale bar: 5 µm. ***F***, Quantification of the number of spines per 20 µm. Data are presented as the mean ± SEM *n* = 18 each; ***p < 0.001; n.s., not significant, Kruskal–Wallis test, Bonferroni *post hoc* test.

As mentioned above, SYNGAP1 has four isoforms (α1, α2, β, and γ) defined according to the alternative splicing between exons 17, 18, and 19. Because of the complexity of the isoform patterns, no study to date has evaluated all the isoforms simultaneously (Araki et al., 2020). To analyze how *SYNGAP1* variants change the alternative splicing of ORF, we performed fragment analysis of *SYNGAP1*-ORF between exon 17, 18, and 19 (Fig. 3*A*). Unexpectedly, we found a significant depletion of the isoform γ in the rs149438267 homozygous motor neurons, which could not be evaluated by Western blotting because of the lack of a specific antibody (Fig. 3*B*; Extended Data Fig. 3-1*A*,*B*). While a significant increase in isoform α1 RNA levels in homozygous neurons was observed at the protein level (Fig. 2*E*), isoform α1 RNA levels did not significantly increase in some lines (Fig. 3*B*; Extended Data Fig. 3-1*A*,*B*).The isoform γ has also been found in mice brain (Araki et al., 2020); however, its function in spine formation has not been elucidated (Kilinc et al., 2018). Interestingly, the overexpression of the SYNGAP1 isoform γ rescued a loss of dendritic spines in rs149438267 homozygous motor neurons (Fig. 3*C–F*), indicating that the SYNGAP1 isoform γ is important for spine formation in motor neurons and can ameliorate spine abnormality in rs149438267 homozygous motor neurons, even in the presence of excess isoform α1. These results suggest that the aberrant splicing, an increase in isoform α1 and a decrease in isoform γ, cause spine loss in the rs149438267 homozygous motor neurons.

### The *SYNGAP1* 3′UTR mutation excessively recruited FUS and HNRNPK

**Figure 4.**
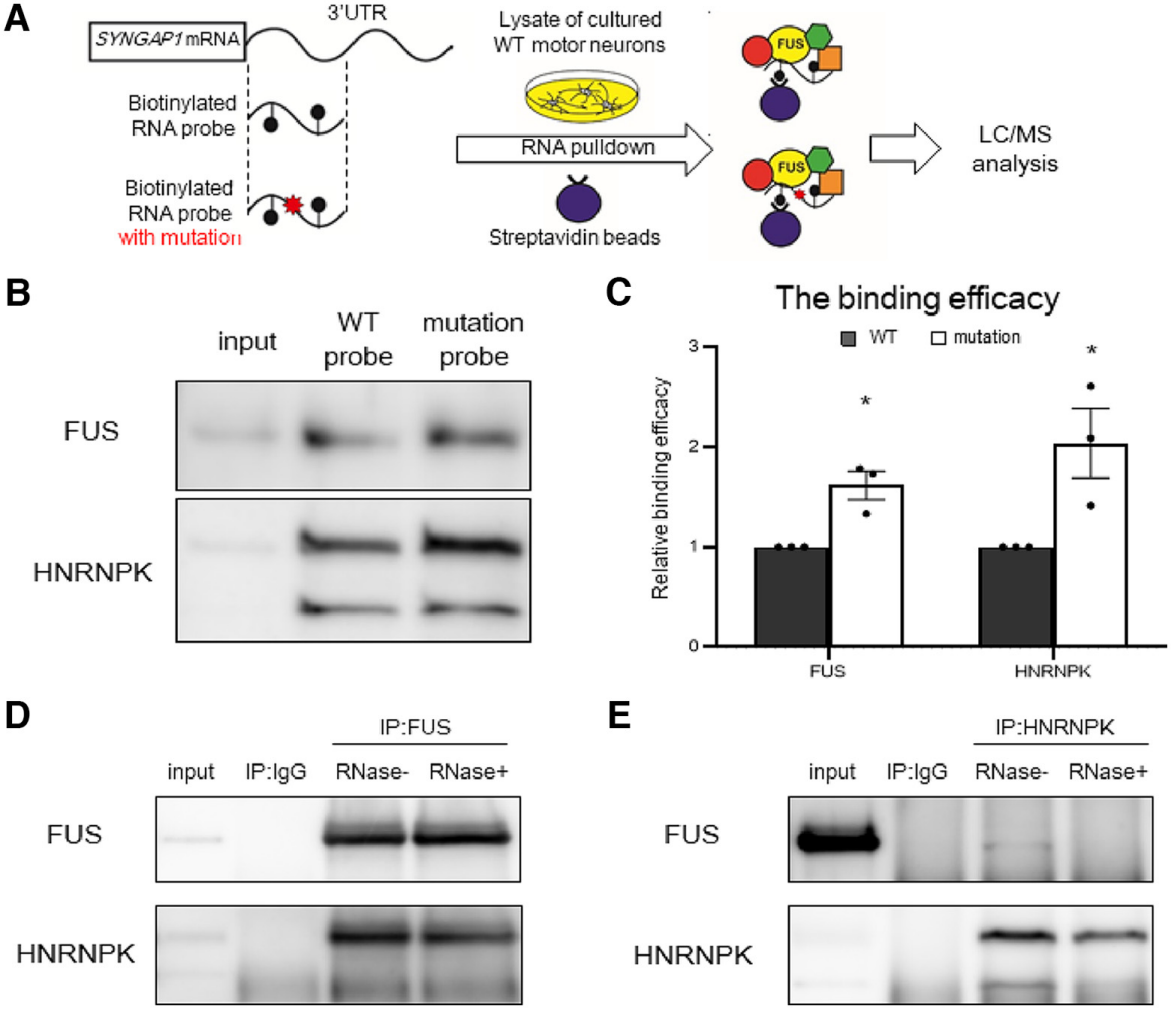
The *SYNGAP1* 3′UTR mutation excessively recruits FUS and HNRNPK. ***A***, A scheme of the biotinylated RNA pull-down assay using *SYNGAP1* mRNA 3′UTR sequences followed by mass spectrometry analysis. ***B***, RNA pull-down assay was performed with a biotinylated RNA probe cloned from the *SYNGAP1* 3′UTR. Pull-down samples were analyzed by Western blotting using the indicated antibodies. ***C***, Quantification of the band intensities in ***B***. Data are presented as the mean ± SEM *n* = 3; *p < 0.05, unpaired *t* test. Additional data of pull-down assay and HNRNPK protein expression are displayed in Extended Data Figure 4-1 and Extended Data Table 4-1. ***D***, ***E***, Co-immunoprecipitation (Co-IP) of FUS (***D***) and HNRNPK (***E***) using lysates from wild-type motor neurons with or without RNase A treatment. The bound proteins were detected with Western blotting and the indicated antibodies. Representative data from triplicate experiments are shown.

To clarify how the *SYNGAP1* 3′UTR variant rs149438267 affects the aberrant splicing of *SYNGAP1* isoforms α1 and γ, we next evaluated the binding efficacy of FUS and collaborating RNA-binding proteins. We generated biotinylated RNA probes with 240-nt sequences for the 5′ side of *SYNGAP1* 3′UTR with or without the mutation (Fig. 4*A*), and performed RNA pull-down assay to analyze the binding efficacy of RNA-binding proteins to each probe by Western blotting. Interestingly, the binding efficacy of FUS to the probe with the mutation was increased compared with the probe without the mutation (Fig. 4*B*,*C*), suggesting that the *SYNGAP1* 3′UTR variant rs149438267 recruited FUS to 3′UTR. In addition to FUS, gel digestion and LC/MS analysis of the specific bands in each sample were performed to examine the proteins that bind to the *SYNGAP1* 3′UTR (Extended Data Fig. 4-1*A*; Extended Data Table 4-1). We found that the binding efficacy of heterogeneous nuclear ribonucleoprotein K (HNRNPK) also increased with the mutated probe (Fig. 4*B*,*C*). HNRNPK is a DNA/RNA-binding protein that plays important roles in transcription (Michelotti et al., 1996; Wang et al., 2020), paraspeckle formation (Fox et al., 2018), splicing (Liu et al., 2018; Thompson et al., 2018), and post-transcriptional regulation at 3′UTR (Yano et al., 2005; Liepelt et al., 2014; Shin et al., 2017). The relationships between HNRNPK and diseases such as cancer (Barboro et al., 2014), Kabuki-like syndrome (Dentici et al., 2018), and Au-Kline syndrome (Au et al., 2018) have been described. The anti-HNRNPK antibody recognizes two splice variants (Barboro et al., 2009). We also analyzed other candidates found in the LC/MS analysis, including ELAVL2 and PUF60, which remained unchanged (Extended Data Fig. 4-1*B*,*C*). Importantly, HNRNPK is also known to interact with FUS (Groen et al., 2013). The expression level of HNRNPK remained unchanged between the WT and the rs149438267 homozygous motor neurons (Extended Data Fig. 4-1*D*,*E*). To validate whether FUS and HNRNPK bind to *SYNGAP1* mRNA, we performed RNA-IP of the motor neuron cultures using the antibodies for each RNA-binding protein. The binding efficacy of *SYNGAP1* mRNA to FUS and HNRNPK tended to be higher in the rs149438267 homozygous motor neurons than in the WT motor neurons (Extended Data Fig. 4-1*F–I*). Moreover, co-immunoprecipitation assay (Co-IP) by the anti-FUS antibody with the RNase A treatment revealed that FUS and HNRNPK bound with each other via protein-protein interactions (Fig. 4*D*). Co-IP by anti-HNRNPK antibody showed that the level of FUS bound to HNRNPK was low and reduced by RNase A treatment. This suggests that HNRNPK is not always bound to FUS, and that some of the FUS proteins bind to HNRNPK via protein–protein interactions and others bind indirectly to HNRNPK via RNA. These results suggest that HNRNPK is a crucial collaborator of FUS, and that both proteins are excessively recruited by the *SYNGAP1* 3′UTR variant.

### HNRNPK, rather than FUS, altered the expression pattern of *SYNGAP1* isoforms

**Figure 5.**
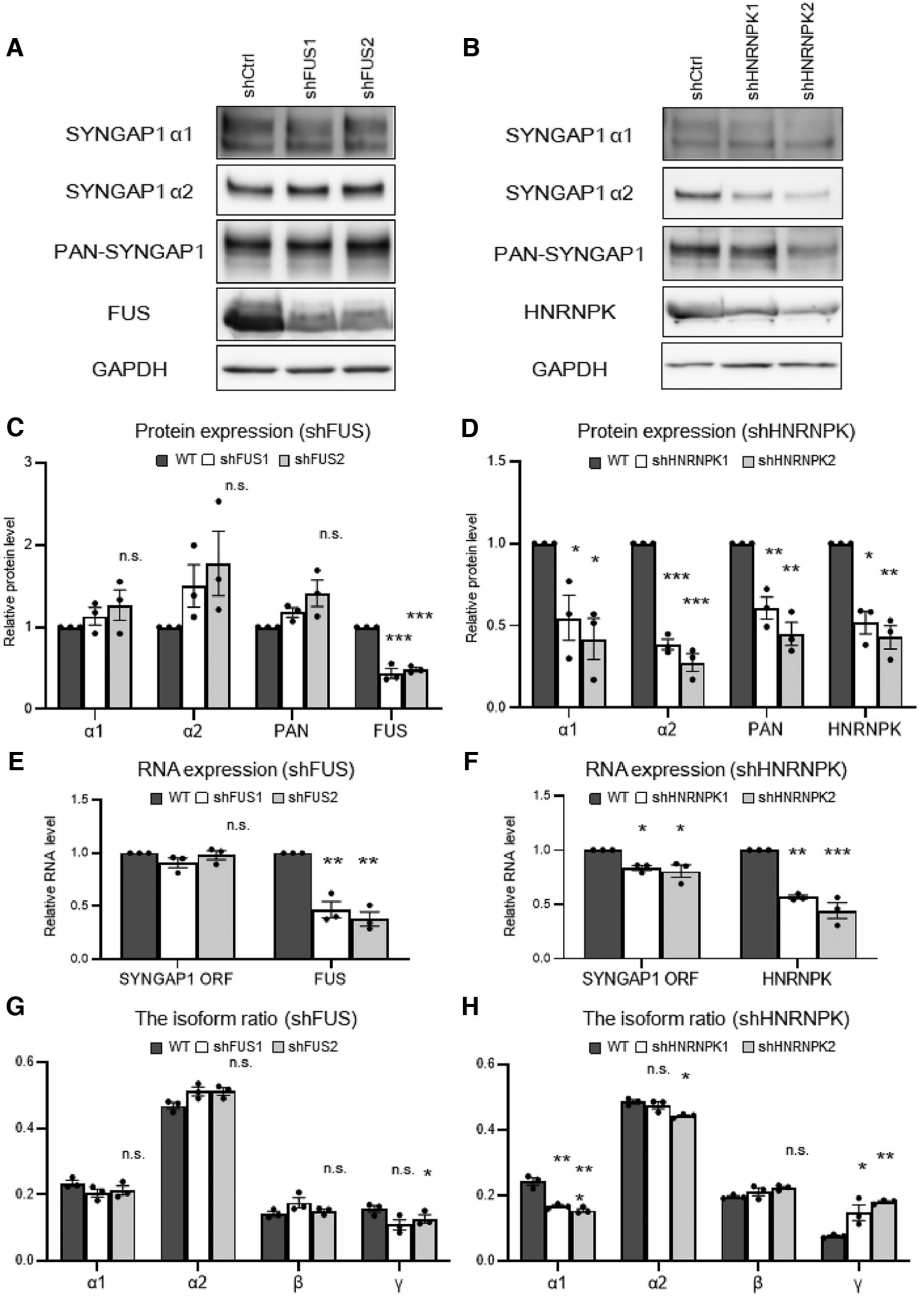
HNRNPK, rather than FUS, alters *SYNGAP1* isoforms similar to those observed in motor neurons with the rs149438267 homozygous mutation. ***A***, ***B***, The lysates from wild-type motor neurons infected with shCtrl, shFUS1, and shFUS2 (***A***) or shCtrl, shHNRNPK1, and shHNRNPK2 (***B***; *n* = 3) were subjected to Western blotting with the indicated antibodies. ***C***, ***D***, Quantification of the band intensities of the indicated proteins in ***A***, ***B***. Data are presented as the mean ± SEM *n* = 3; *p < 0.05, **p < 0.01, ***p < 0.001, one-way ANOVA, Tukey's *post hoc* test. ***E***, ***F***, Total RNA was extracted from wild-type motor neurons infected with shCtrl, shFUS1, and shFUS2 (***E***) or shCtrl, shHNRNPK1, and shHNRNPK2 (***F***; *n* = 3 neuron cultures each), and the mRNA expression levels of *SYNGAP1*, *FUS*, and *HNRNPK* were analyzed with qRT-PCR. Data are presented as the mean ± SEM; *p < 0.05, **p < 0.01, ***p < 0.001, one-way ANOVA, Tukey's *post hoc* test. ***G***, ***H***, Total RNA from wild-type motor neurons infected with shCtrl, shFUS1, and shFUS2 (***G***; *n* = 4) or shCtrl, shHNRNPK1, and shHNRNPK2 (***H***; *n* = 3) was analyzed with RT-PCR and fragment analysis to evaluate the change in the *SYNGAP1* isoform ratio. Data are presented as the mean ± SEM; *p < 0.05, **p < 0.01, ***p < 0.001, one-way ANOVA, Tukey's *post hoc* test. Additional data of altered *SYNGAP1* splicing by HNRNPK are displayed in Extended Data Figure 5-1.

Next, we performed knock-down experiments to elucidate how FUS and HNRNPK regulate *SYNGAP1* isoforms. It should be noted that after HNRNPK knock-down, motor neurons could not survive for four weeks, and thus we collected samples on day 14. Western blot analysis (Fig. 5*A–D*) showed that FUS knock-down did not result in significant changes in SYNGAP1 expression levels (Fig. 5*C*). More importantly, HNRNPK knock-down decreased PAN-SYNGAP1 as well as isoforms α1 and α2 (Fig. 5*D*). qRT-PCR revealed that HNRNPK knock-down, but not FUS knock-down, decreased the total expression of *SYNGAP1*-ORF (Fig. 5*E*,*F*), consistent with the results of Western blotting (Fig. 5*C*,*D*). Fragment analysis also revealed that HNRNPK knock-down decreased isoform α1 and increased isoform γ levels, while FUS knock-down slightly decreased isoform γ levels (Fig. 5*G*,*H*). HNRNPK knock-down also induced another undescribed alternative splicing variant, which could be translated into isoform γ but had longer intron sequences than the conventional γ sequence (Extended Data Fig. 5-1*A*). These results indicate HNRNPK-controlled alternative splicing of *SYNGAP1* mRNA, especially isoform γ. In addition, the changes in SYNGAP1 expression or isoform alteration in the double-knock-down of FUS and HNRNPK were similar to those in the HNRNPK knock-down model (Extended Data Fig. 5-1*B–E*), which suggests that HNRNPK plays a major role in controlling SYNGAP1 expression. FUS had no significant effect on the transcript levels of SYNGAP1 variants, but it affected SYNGAP1 α1 protein expression, which was decreased in the HNRNPK knock-down but not in the double-knock-down. Given that the *SYNGAP1* variant rs149438267 caused aberrant splicing, thus increasing isoform α1 and decreasing isoform γ, these results indicate that HNRNPK, rather than FUS, might contribute to the altered splicing of *SYNGAP1* isoforms through the variant. However, the knock-down experiments could not directly explain the relationship between the excessive recruitment of RNA-binding proteins, aberrant splicing, and dendritic spine loss.

### The antisense oligonucleotides toward the HNRNPK binding sites ameliorated spine abnormalities

**Figure 6.**
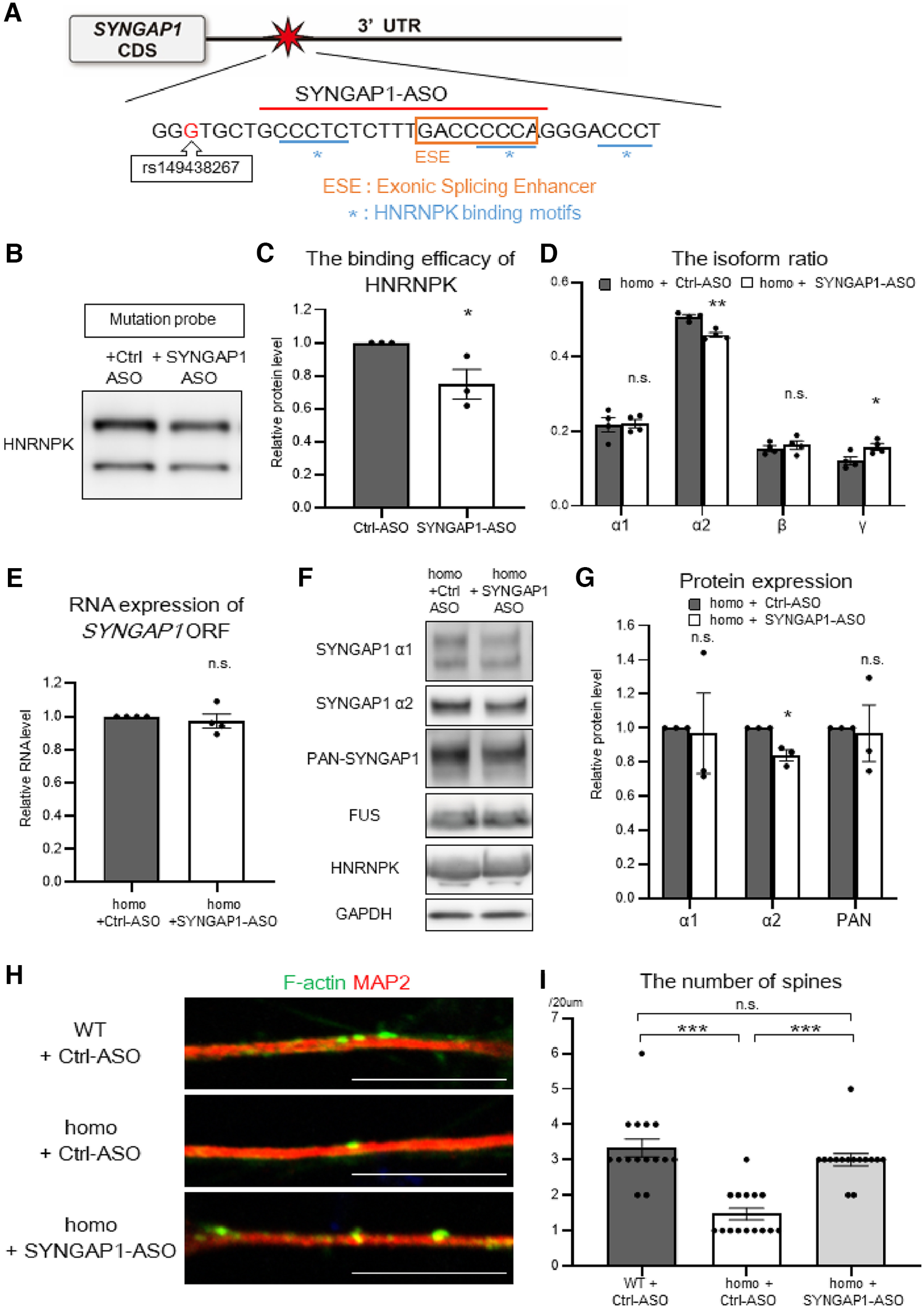
Antisense oligonucleotides blocked the HNRNPK binding site and ameliorated a loss of dendritic spines. ***A***, A schematic overview of antisense oligonucleotides toward *SYNGAP1* 3′UTR. The scheme shows the sequences representing the binding motifs of HNRNPK according to the mCrossBase database (underlined in blue) and the exonic splicing enhancer according to the RegRNA database (orange frame). ***B***, RNA pull-down assay was performed with the lysates from the wild-type motor neurons and biotinylated RNA probe with rs149438267. Pull-down samples were analyzed by Western blotting using the indicated antibodies. ***C***, Quantification of the band intensities in ***B***. Data are presented as the mean ± SEM *n* = 3 each; *p < 0.05, unpaired *t* test. ***D***, Fragment analysis of RNA extracted from motor neurons with the homozygous mutation 14 d after Ctrl-ASO or SYNGAP1-ASO treatment. Data are presented as the mean ± SEM *n* = 4 each; **p < 0.01, *p < 0.05; n.s., not significant, unpaired *t* test. ***E***, RNA samples (same as in ***D***) were analyzed with RT-PCR and the primer set for *SYNGAP1*-ORF. Data are presented as the mean ± SEM *n* = 4 each; unpaired *t* test. ***F***, Motor neurons with the homozygous mutation were treated with Crtl-ASO or SYNGAP1-ASO for 14 d and were then subjected to Western blotting with the indicated antibodies. ***G***, Quantification of the band intensities of the proteins in ***F***. Data are presented as the mean ± SEM *n* = 3; *p < 0.05; n.s., not significant, unpaired *t* test. ***H***, Motor neurons were immunostained for F-actin (green) and MAP2 (red). Scale bars: 10 µm. ***I***, Quantification of the number of spines per 20 µm. Data are presented as the mean ± SEM *n* = 15 each; ***p < 0.001, n.s. not significant, Kruskal–Wallis test, Bonferroni *post hoc* test. The fundamental data of SYNGAP1-ASO toward the binding site of HNRNPK are displayed in Extended Data Figure 6-1.

To further elucidate how the excessive recruitment of HNRNPK contributes to the aberrant splicing and dendritic spine loss in the rs149438267 homozygous motor neurons, we considered blocking the recruitment of RNA-binding proteins with antisense oligonucleotides (ASOs), which may represent a therapeutic reagent for ALS (Kole et al., 2012; Bennett et al., 2019). According to the eCLIP of K562 cells from the ENCODE datasets (Davis et al., 2018), the *SYNGAP1* 3′UTR variant rs149438267 was located between the putative FUS and HNRNPK binding sites (Extended Data Fig. 6-1*A*). Moreover, the RegRNA database showed that the exonic splicing enhancer was present at the 3′ side of the variant (Chang et al., 2013), and the mCrossBase database showed that the HNRNPK binding motifs were distributed around the exonic splicing enhancer (Feng et al., 2019; Fig. 6*A*).

Referring to these data, we designed three ASOs toward *SYNGAP1* 3′UTR (E1, E2, and E3) and treated the rs149438267 homozygous motor neurons with 10 or 50 nm of these ASOs, followed by evaluation after 14 d (Extended Data Fig. 6-1*B*). We then selected E2-ASO as SYNGAP1-ASO, which exhibited an increase of *SYNGAP1* isoform γ at 50 nm, as observed using fragment analysis in a single experiment (Extended Data Fig. 6-1*C*,*D*). Pull-down assay confirmed that SYNGAP1-ASO could block the excessive binding of HNRNPK to the probe with the *SYNGAP1* 3′UTR mutation (Fig. 6*B*,*C*). Unexpectedly, SYNGAP1-ASO significantly increased the binding efficacy of FUS to the mutation probe (Extended Data Fig. 6-1*E*,*F*). Moreover, pull-down assay of HNRNPK knock-down showed that the depletion of HNRNPK did not increase FUS binding to the mutation probe (Extended Data Fig. 6-1*G*,*H*), which suggests that HNRNPK and FUS interact indirectly at *SYNGAP1* 3′UTR, and that ASO independently blocked HNRNPK and increased FUS binding. Next, we analyzed the effects of SYNGAP1-ASO on the expression pattern of the *SYNGAP1* isoforms. Fragment analysis revealed that the treatment of SYNGAP1-ASO for 14 d in the rs149438267 homozygous motor neurons increased the expression ratio of isoform γ, decreased the expression ratio of isoform α2, and did not alter the expression ratio of isoform α1 (Fig. 6*D*). qRT-PCR revealed that the expression level of total *SYNGAP1* was not affected by SYNGAP1-ASO (Fig. 6*E*), suggesting that SYNGAP1-ASO could solely affect the alternative splicing of *SYNGAP1* isoforms. In addition, SYNGAP1-ASO did not alter the *SYNGAP1* isoforms in WT neurons 14 d after ASO treatment (Extended Data Fig. 6-1*G*,*H*), suggesting that SYNGAP1-ASO could specifically correct the excessive recruitment of HNRNPK induced by the variant. Western blotting showed that the expression of SYNGAP1 α2 was decreased in the rs149438267 homozygous motor neurons 14 d after SYNGAP1-ASO treatment, while SYNGAP1 α1 and PAN-SYNGAP1 remained unchanged (Fig. 6*F*,*G*), consistent with the results of fragment analysis (Fig. 6*D*).

**Figure 7.**
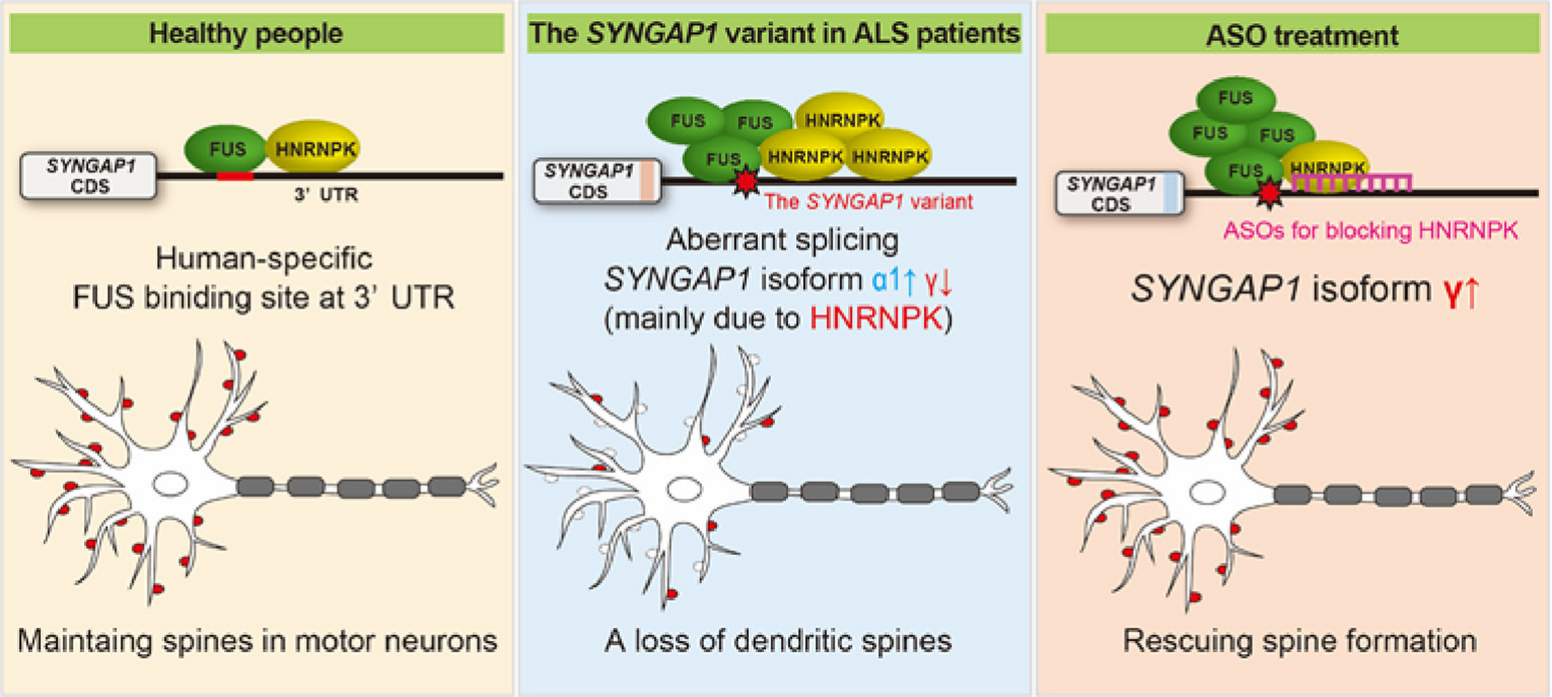
The *SYNGAP1* 3′UTR variant excessively recruits HNRNPK, causing aberrant *SYNGAP1* splicing and dendritic spine loss. A graphical summary of the mechanism of dendritic spine loss by the *SYNGAP1* 3′UTR variant. The *SYNGAP1* 3′UTR variant excessively recruits RNA-binding proteins, causing aberrant *SYNGAP1* splicing and dendritic spine loss. ASOs that block HNRNPK recruitment increase expression of the SYNGAP1 isoform γ and ameliorate the loss of dendritic spines, which suggests that HNRNPK plays an important role in dendritic spine loss of motor neurons with the *SYNGAP1* 3′UTR variant.

Finally, SYNGAP1-ASO treatment for 14 d could ameliorate a loss of dendritic spines in the rs149438267 homozygous motor neurons (Fig. 6*H*,*I*). This suggests that the excessive recruitment of HNRNPK was the major pathogenic mechanism for dendritic spine loss in the rs149438267 homozygous motor neurons. Together, these results clarify that the *SYNGAP1* 3′UTR variant rs149438267 identified in the Japanese ALS cohort excessively recruits RNA-binding proteins, especially HNRNPK, and causes a loss of dendritic spines in iPSC-derived motor neurons (Fig. 7).

## Discussion

In this study, we screened ALS patients in a combined Japanese cohort at the FUS binding site in the *SYNGAP1* 3′UTR. We found the variant rs149432867 was associated with a loss of dendritic spines in iPSC-derived motor neurons. While previous studies have demonstrated that *SYNGAP1* is a pathogenic candidate for autism spectrum disorder (Mignot et al., 2016), no report to date has demonstrated a relationship between *SYNGAP1* and ALS. Interestingly, *SYNGAP1* mutations in individuals with autism spectrum disorder represent loss-of-function mutations (Mignot et al., 2016), and *Syngap1* hetero knock-out mice show an increase in the proportion of mature spines (Clement et al., 2012). Our findings of spine loss by *SYNGAP1* 3′UTR variant rs149432867 differ from the function of *SYNGAP1* observed in previous research. Synapse loss has been reported as an early pathologic feature of neurodegenerative diseases, including ALS (Sasaki and Maruyama, 1994; Herms and Dorostkar, 2016; Henstridge et al., 2018). Also, synaptic dysfunction has been reported to contribute to early motor and cognitive dysfunctions before neuronal death in a mouse model of ALS/FTLD (Sunico et al., 2011; Yokoi et al., 2017). In this study, we confirm the presence of dendritic spine loss by the *SYNGAP1* 3′UTR rs149438267 in iPSC-derived homozygous motor neurons, which could help to elucidate the pathogenesis of early-stage ALS.

We found that the *SYNGAP1* 3′UTR rs149438267 variant excessively recruited FUS and HNRNPK. Interestingly, the cooperative protein with FUS is quite different from that in mice (Yokoi et al., 2017). Moreover, we found that ASO, which blocks the excessive recruitment of HNRNPK to *SYNGAP1* 3′UTR, could alter the *SYNGAP1* splicing, and ameliorate dendritic spine loss in the rs149432867 homozygous motor neurons. This result emphasizes that excess HNRNPK recruitment is the crucial mechanism underlying dendritic spin formation in motor neurons. *SYNGAP1* 3′UTR contains exonic splicing enhancers close to the HNRNPK binding site, and HNRNPK is known to affect the association between RNA-binding proteins and exonic splicing enhancers or silencers (Marchand et al., 2011). The variants at the 3′UTR have been reported to affect the alternative splicing of ORF (Zhao et al., 2019). Because of such splicing machinery, the excessive binding of HNRNPK itself or HNRNPK and other collaborating RNA-binding proteins could have introduced the aberrant splicing in the rs149432867 homozygous motor neurons.

We also found that HNRNPK affected the SYNGAP1 isoform γ. SYNGAP1 has four isoforms, of which isoform α1 is the most validated isoform that has a PDZ domain that interacts with proteins at the postsynaptic density and regulates synaptic plasticity (Kim et al., 1998; Araki et al., 2015). There have been few reports on the function of SYNGAP1 isoform γ (Araki et al., 2020). Our findings indicate that the SYNGAP1 isoform γ plays an important role in spine formation in human models. While the SYNGAP1 isoform γ was increased, α1 remained unchanged, and α2 was decreased by ASO treatment. Together with the results of double-knock-down of FUS and HNRNPK, this indicates that FUS affects HNRNPK-dependent alternation of the SYNGAP1 α1 level. Although the decrease in the SYNGAP1 isoform α2 might have suppressed synaptic strength (McMahon et al., 2012), the decrease in α1 and increase in γ improved spine abnormality, possibly by overcoming the effects of α2. Also, ASO could ameliorate spine loss without sequestering FUS from *SYNGAP1* 3′UTR by ASO. These data indicate that HNRNPK regulation of the SYNGAP1 isoform γ, which is independent of FUS and SYNGAP1 isoform α1, is a crucial mechanism underlying spine abnormality caused by the *SYNGAP1* 3′UTR rs149438267 variant. Further evaluations in motor neurons with FUS mutation are needed to elucidate whether FUS has the pathogenic correlation with *SYNGAP1*.

In this study, there were some discrepancies between the Western blot and fragment analysis results in the evaluation of SYNGAP1 α isoforms. These could be a result of translational control of *SYNGAP1* mRNA (Popovitchenko et al., 2020) or isoform-specific protein stability control (Vuong et al., 2022). Since there was a difference of only one base pair between isoform α1 and α2, it is difficult to distinguish each mRNA to analyze translational control. Moreover, given that SYNGAP1 isoforms are defined within a long region of alternative splicing between exons 17, 18, and 19, it is difficult to evaluate all isoforms simultaneously, even with next-generation sequencing (Araki et al., 2020). These discrepancies might be also because of the insufficient detection capability of fragment analysis. In addition, protein expression of isoforms β and γ could not be analyzed because of the absence of specific antibodies. Further developments in methods to elucidate the regulation of SYNGAP1 isoforms are expected.

We could not confirm aberrant *SYNGAP1* alterations in the rs149432867 heterozygous motor neurons. This might be because of the experimental settings, e.g., the duration of motor neuron culturing or the composition of the motor neuron medium (Bardy et al., 2015; Odawara et al., 2016). In addition, we could not generate iPSCs from patients with the *SYNGAP1* variants rs149432867 because of ethical limitations. We could not obtain informed consent from patients with *SYNGAP1* variant rs149432867 to generate iPSCs from their samples. It should also be noted that the heterozygous variant might have milder pathologies than the homozygous variant. Thus, a more detailed analysis of heterozygous motor neurons is needed to fully demonstrate the relevance of this variant in the pathogenesis of ALS.

In summary, we identified the *SYNGAP1* 3′UTR variant rs149432867 from the Japanese sporadic ALS cohort as a major candidate involved in a loss of dendritic spines in iPSC-derived motor neurons. In addition, we demonstrated that the *SYNGAP1* 3′UTR variant altered *SYNGAP1* isoforms via the excess recruitment of FUS and HNRNPK; in particular, HNRNPK played an important role in *SYNGAP1* splicing and spine formation. Our findings provide a basis for the future exploration of ALS-related RNA-binding proteins.

10.1523/JNEUROSCI.0455-22.2022.f6-1 Extended Data Figure 6-1 The fundamental data of SYNGAP1-ASO toward the binding site of HNRNPK. ***A***, eCLIP data of K562 cells from ENCODE. The *SYNGAP1 3*′UTR variant was located between the binding sites of HNRNPK and FUS. ***B***, A schematic overview of the construction of antisense oligonucleotides (ASOs) for the *SYNGAP1* 3′UTR. ***C***, A schematic overview of the ASO experiments. ***D***, Fragment analysis of RNA extracted from motor neurons with the homozygous mutation 14 d after SYNGAP1-ASO (E1–E3) treatment; NT, not treated; *n* = 1 each. ***E***, RNA pull-down assay was performed under the same conditions as in Figure 6*B*. Pull-down samples were analyzed with Western blotting using the indicated antibodies. ***F***, Quantification of the band intensities in ***E***. *n* = 4; ***p < 0.001, Mann–Whitney *U* test. ***G***, RNA pull-down assay was performed with biotinylated RNA probe with rs149438267 and the lysates from the wild-type motor neurons infected with shCtrl or shHNRNPK. Pull-down samples were analyzed using Western blotting with the indicated antibodies. ***H***, Quantification of the band intensities in ***G***. *n* = 4; ***p < 0.001; n.s., not significant, unpaired *t* test. ***I***, Fragment analysis of RNA extracted from wild-type motor neurons treated with Ctrl-ASO or SYNGAP1-ASO for 14 d. Data are presented as the mean ± SEM *n* = 3; n.s., not significant, unpaired *t* test. ***J***, qRT-PCR with the primer set for *SYNGAP1*-ORF was performed with the same RNA samples as in ***I***. Data are presented as the mean ± SEM *n* = 3; n.s., not significant, unpaired *t* test. Download Figure 6-1, TIF file.

10.1523/JNEUROSCI.0455-22.2022.f5-1 Extended Data Figure 5-1 HNRNPK altered *SYNGAP1* splicing by intron retention. ***A***, Total RNA from wild-type motor neurons infected with shCtrl, shHNRNPK1, and shHNRNPK2 was analyzed with RT-PCR (as in Fig. 5*H*). The samples were subjected to agarose electrophoresis. The upper band was analyzed by Sanger sequencing. Of note, an alternative splicing variant of the *SYNGAP1* isoform γ containing a longer intron sequence (red) was observed. ***B***, Total RNA was extracted from wild-type motor neurons infected with shCtrl or shFUS + shHNRNPK (*n* = 4 neuron cultures each), and the mRNA expression levels of *SYNGAP1*, *FUS*, and *HNRNPK* were analyzed using qRT-PCR. Data are presented as the mean ± SEM; *p < 0.05, **p < 0.01, ***p < 0.001, unpaired *t* test. ***C***, Total RNA from wild-type motor neurons infected with shCtrl or shFUS + shHNRNPK (*n* = 3 each) was analyzed using RT-PCR and fragment analysis to evaluate the change in the *SYNGAP1* isoform ratio. Data are presented as the mean ± SEM; *p < 0.05, **p < 0.01; n.s., not significant, unpaired *t* test. ***D***, The lysates from wild-type motor neurons infected with shCtrl or shFUS + shHNRNPK (*n* = 3 each) were subjected to Western blotting with the indicated antibodies. ***E***, Quantification of the band intensities of the indicated proteins in ***D***. Data are presented as the mean ± SEM *n* = 3; *p < 0.05; n.s., not significant, unpaired *t* test. Download Figure 5-1, TIF file.

10.1523/JNEUROSCI.0455-22.2022.f4-1 Extended Data Figure 4-1 Pull-down assay and HNRNPK protein expression. ***A***, RNA pull-down assay was performed using biotinylated RNA probes cloned from the *SYNGAP1 3*′UTR. Pull-down samples were analyzed with silver staining. The specific bands that were cut and subjected to LC/MS analysis are shown in each lane (white triangles). ***B***, RNA pull-down samples were subjected to Western blotting with the indicated antibodies. ***C***, Quantification of the band intensities in ***B***. Data are presented as the mean ± SEM *n* = 3; n.s., not significant, unpaired *t* test. ***D***, The lysates from wild-type and heterozygous iPSC-derived motor neurons were analyzed with Western blotting and the indicated antibodies. ***E***, Quantification of the band intensities of the indicated proteins in ***D***. *n* = 3; n.s., not significant, one-way ANOVA, Tukey's *post hoc* test. ***F***, ***H***, RNA-immunoprecipitation (IP) of FUS (F) and HNRNPK (H) in motor neurons with wild-type or homozygous mutations. The bound RNA was analyzed with qRT-PCR using the primer sets indicated in the graph. The IP efficiency was calculated relative to the input. Representative data from triplicate experiments are shown. ***G***, ***I***, The FUS-IP (***G***) and HNRNPK-IP (***I***) efficiency of *SYNGAP1* mRNA in the homozygous motor neurons in ***F***, ***H*** was calculated relative to that in wild-type motor neurons. *n* = 3 each; unpaired *t* test. Download Figure 4-1, TIF file.

10.1523/JNEUROSCI.0455-22.2022.f3-1 Extended Data Figure 3-1 The isoform changes in motor neurons with *SYNGAP1* homozygous mutation. ***A***, ***B***, RNA from wild-type and homozygous 2 (homo2; ***A***) or homozygous 3 (homo3; ***B***) motor neurons were analyzed with RT-PCR, and the PCR products were analyzed with fragment analysis. Data are presented as the mean ± SEM *n* = 3 for homo2, *n* = 6 for homo3; *p < 0.05, **p < 0.01, ***p < 0.001; n.s., not significant, unpaired *t* test. Download Figure 3-1, TIF file.

10.1523/JNEUROSCI.0455-22.2022.f2-1 Extended Data Figure 2-1 *SYNGAP1* 3′UTR in iPSC-derived motor neurons. ***A***, RNA from wild-type motor neurons was analyzed by 3′RACE and agarose gel electrophoresis. Note that short 3′UTR was present below the full-length 3′UTR. ***B***, The bands in ***A*** were extracted and analyzed by Sanger sequencing to identify the ORF splicing patterns; full-length 3′UTR (*n* = 43); short 3′UTR (*n* = 38); n.s., not significant, χ^2^ test. ***C***, The 3′UTR splicing patterns in ***B***. The lengths of full-length 3′UTR (red line) and short 3′UTR (black lines). The spaces between the black lines represent the sites of 3′UTR skipping. ***D***, RT-PCR was performed using the indicated primer sets to amplify DNA extracted from wild-type and edited heterozygous iPSCs. PCR products were digested by Msl1, a noncutter for the *SYNGAP1* mutation, and analyzed with agarose gel electrophoresis. ***E***, The lysates from wild-type and heterozygous iPSC-derived motor neurons were analyzed with Western blotting using the indicated antibodies. ***F***, Quantification of the band intensities of the indicated proteins in ***E***. *n* = 3; n.s., not significant, one-way ANOVA, Tukey's *post hoc* test. Download Figure 2-1, TIF file.

10.1523/JNEUROSCI.0455-22.2022.f1-1 Extended Data Figure 1-1 The FUS binding sites at *SYNGAP1* 3′UTR and induced pluripotent stem cell (iPSC)-derived motor neurons. ***A***, FUS CLIP-seq data from the temporal lobe of three epilepsy patients who underwent lobectomy (Nakaya et al., 2013). The number represents a pile-up of CLIP tags. ***B***, The DNA sequence of iPSCs with the homozygous mutation (homo1). ***C***, A schematic overview of the differentiation and maturation of iPSC-derived motor neurons from embryoid bodies (EBs). ***D***, iPSC-derived motor neurons with the wild-type or homozygous mutation that were cultured for one week from EBs were immunostained for ISL-1 (green), HB9 (red), βIII-tubulin (white), and DAPI (blue). Scale bars: 10 µm. ***E***, Quantification of the ratio of ISL-1/βIII-tubulin-positive and HB9/βIII-tubulin-positive cells per DAPI-positive cells. Data are presented as the mean ± SEM *n* = 6 fields from 3 independent wells; n.s., not significant, one-way ANOVA, Tukey's *post hoc* test (ISL-1), Kruskal–Wallis test, Bonferroni *post hoc* test (HB9). ***F***, iPSC-derived motor neurons were immunostained for F-actin (green), MAP2 (red), and Synaptophysin (white). Scale bar: 5 µm. ***G***, Quantification of the number of spines per 20 µm of dendrite length. Data are presented as the mean ± SEM *n* = 16 each from triplicate experiments; ***p < 0.001, Kruskal–Wallis test, Bonferroni *post hoc* test. Download Figure 1-1, TIF file.

10.1523/JNEUROSCI.0455-22.2022.tab4-1 Extended Data Table 4-1 LC/MS/MS results of proteins from the pull-down assay in Extended Data Figure 4-1*A* (provided in Excel files). Download Table 4-1, XLSX file.
