# Supplementary figures and images for: The SYNGAP1 3′UTR Variant in ALS Patients Causes Aberrant SYNGAP1 Splicing and Dendritic Spine Loss by Recruiting HNRNPK
Source: J Neurosci. 2022 Nov 23;42(47):8881–96. doi: 10.1523/JNEUROSCI.0455-22.2022 (PMC9698725; doi:10.1523/JNEUROSCI.0455-22.2022)

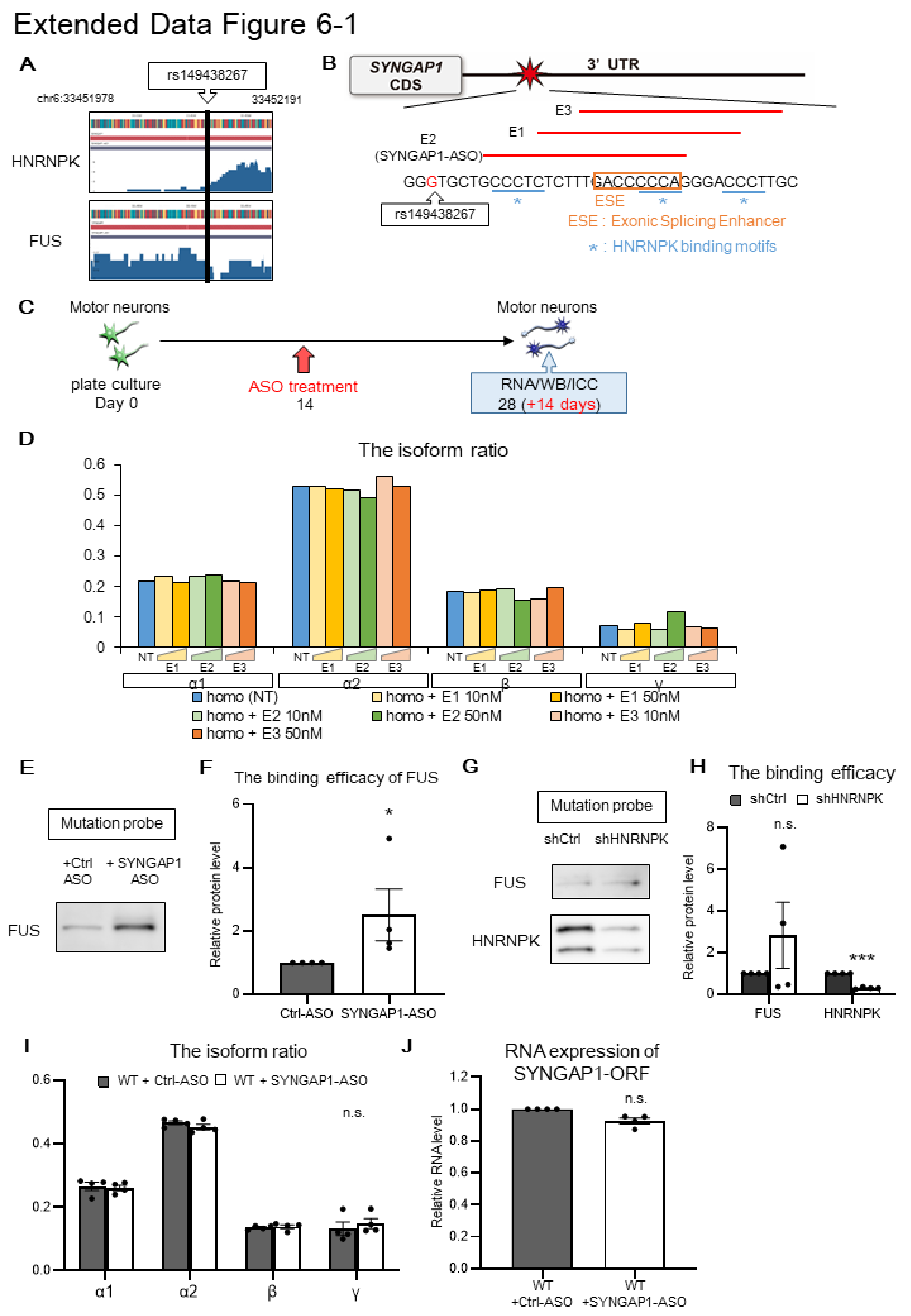

Supplement: Extended Data Figure 6-1 — The fundamental data of SYNGAP1-ASO toward the binding site of HNRNPK. A, eCLIP data of K562 cells from ENCODE. The SYNGAP1 3′UTR variant was located between the binding sites of HNRNPK and FUS. B, A schematic overview of the construction of antisense oligonucleotides (ASOs) for the SYNGAP1 3′UTR. C, A schematic overview of the ASO experiments. D, Fragment analysis of RNA extracted from motor neurons with the homozygous mutation 14 d after SYNGAP1-ASO (E1–E3) treatment; NT, not treated; n = 1 each. E, RNA pull-down assay was performed under the same conditions as in Figure 6B. Pull-down samples were analyzed with Western blotting using the indicated antibodies. F, Quantification of the band intensities in E. n = 4; ***p < 0.001, Mann–Whitney U test. G, RNA pull-down assay was performed with biotinylated RNA probe with rs149438267 and the lysates from the wild-type motor neurons infected with shCtrl or shHNRNPK. Pull-down samples were analyzed using Western blotting with the indicated antibodies. H, Quantification of the band intensities in G. n = 4; ***p < 0.001; n.s., not significant, unpaired t test. I, Fragment analysis of RNA extracted from wild-type motor neurons treated with Ctrl-ASO or SYNGAP1-ASO for 14 d. Data are presented as the mean ± SEM n = 3; n.s., not significant, unpaired t test. J, qRT-PCR with the primer set for SYNGAP1-ORF was performed with the same RNA samples as in I. Data are presented as the mean ± SEM n = 3; n.s., not significant, unpaired t test. Download Figure 6-1, TIF file. [file ns-JN-RM-0455-22-s07.tif]

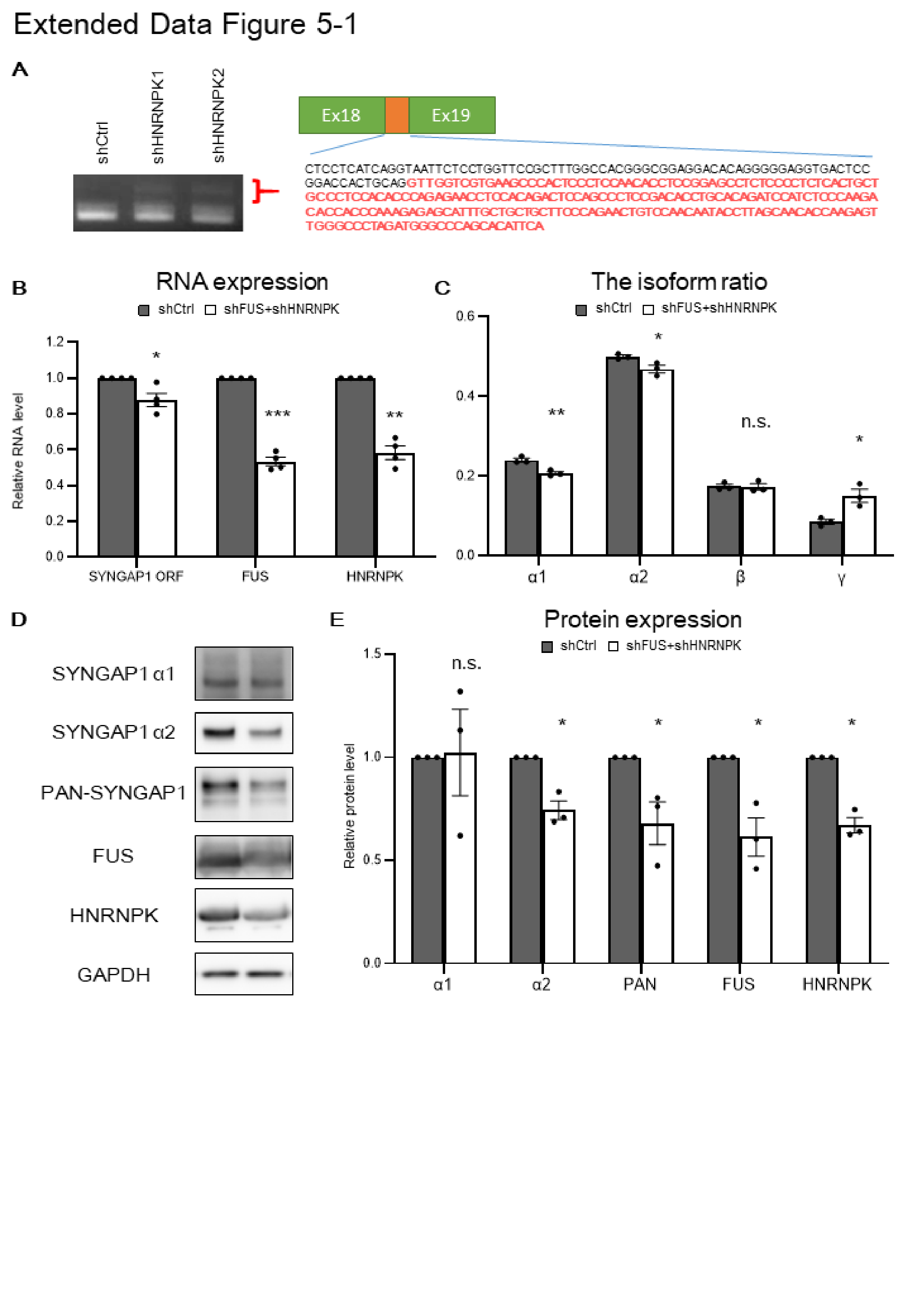

Supplement: Extended Data Figure 5-1 — HNRNPK altered SYNGAP1 splicing by intron retention. A, Total RNA from wild-type motor neurons infected with shCtrl, shHNRNPK1, and shHNRNPK2 was analyzed with RT-PCR (as in Fig. 5H). The samples were subjected to agarose electrophoresis. The upper band was analyzed by Sanger sequencing. Of note, an alternative splicing variant of the SYNGAP1 isoform γ containing a longer intron sequence (red) was observed. B, Total RNA was extracted from wild-type motor neurons infected with shCtrl or shFUS + shHNRNPK (n = 4 neuron cultures each), and the mRNA expression levels of SYNGAP1, FUS, and HNRNPK were analyzed using qRT-PCR. Data are presented as the mean ± SEM; *p < 0.05, **p < 0.01, ***p < 0.001, unpaired t test. C, Total RNA from wild-type motor neurons infected with shCtrl or shFUS + shHNRNPK (n = 3 each) was analyzed using RT-PCR and fragment analysis to evaluate the change in the SYNGAP1 isoform ratio. Data are presented as the mean ± SEM; *p < 0.05, **p < 0.01; n.s., not significant, unpaired t test. D, The lysates from wild-type motor neurons infected with shCtrl or shFUS + shHNRNPK (n = 3 each) were subjected to Western blotting with the indicated antibodies. E, Quantification of the band intensities of the indicated proteins in D. Data are presented as the mean ± SEM n = 3; *p < 0.05; n.s., not significant, unpaired t test. Download Figure 5-1, TIF file. [file ns-JN-RM-0455-22-s06.tif]

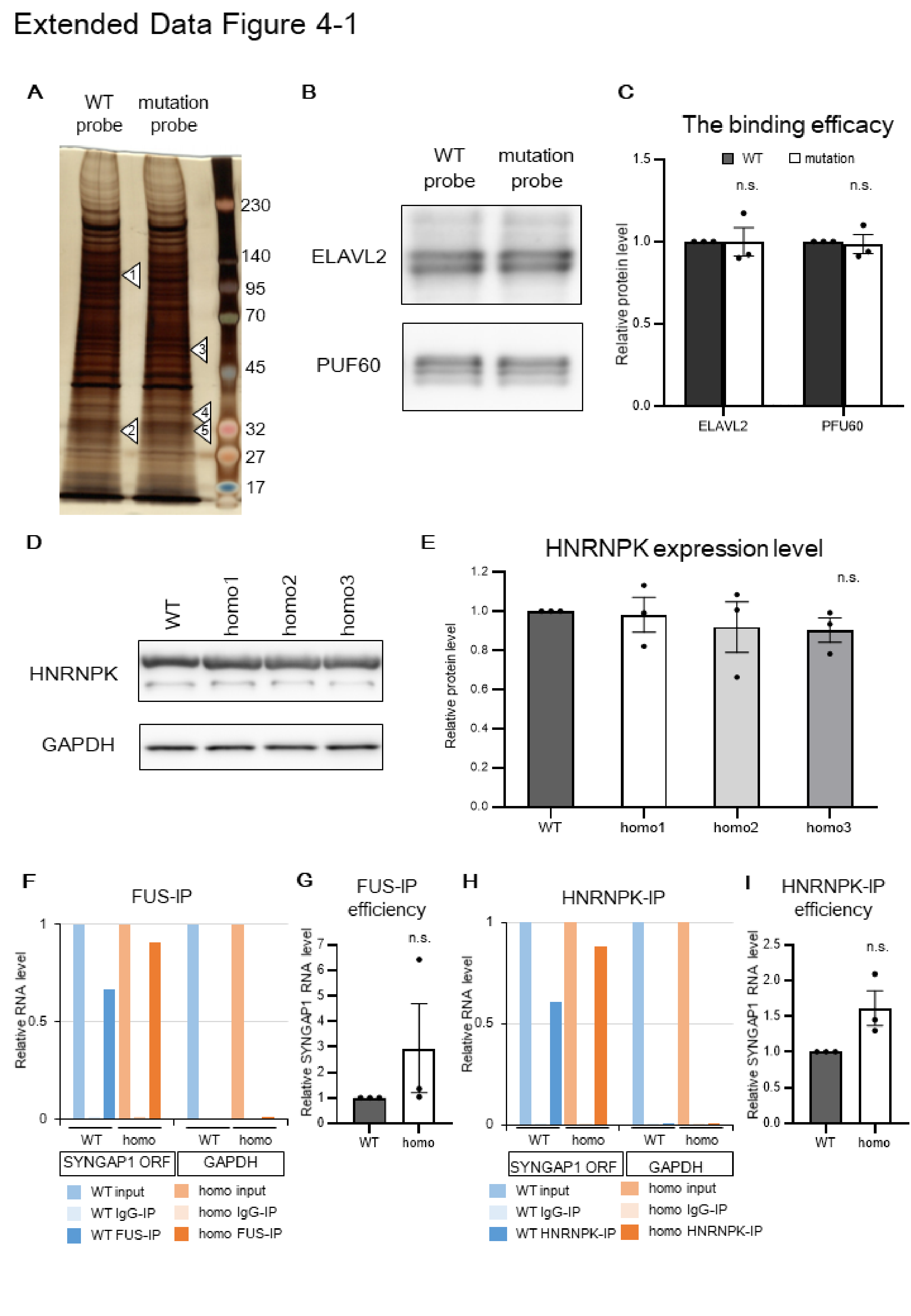

Supplement: Extended Data Figure 4-1 — Pull-down assay and HNRNPK protein expression. A, RNA pull-down assay was performed using biotinylated RNA probes cloned from the SYNGAP1 3′UTR. Pull-down samples were analyzed with silver staining. The specific bands that were cut and subjected to LC/MS analysis are shown in each lane (white triangles). B, RNA pull-down samples were subjected to Western blotting with the indicated antibodies. C, Quantification of the band intensities in B. Data are presented as the mean ± SEM n = 3; n.s., not significant, unpaired t test. D, The lysates from wild-type and heterozygous iPSC-derived motor neurons were analyzed with Western blotting and the indicated antibodies. E, Quantification of the band intensities of the indicated proteins in D. n = 3; n.s., not significant, one-way ANOVA, Tukey's post hoc test. F, H, RNA-immunoprecipitation (IP) of FUS (F) and HNRNPK (H) in motor neurons with wild-type or homozygous mutations. The bound RNA was analyzed with qRT-PCR using the primer sets indicated in the graph. The IP efficiency was calculated relative to the input. Representative data from triplicate experiments are shown. G, I, The FUS-IP (G) and HNRNPK-IP (I) efficiency of SYNGAP1 mRNA in the homozygous motor neurons in F, H was calculated relative to that in wild-type motor neurons. n = 3 each; unpaired t test. Download Figure 4-1, TIF file. [file ns-JN-RM-0455-22-s04.tif]

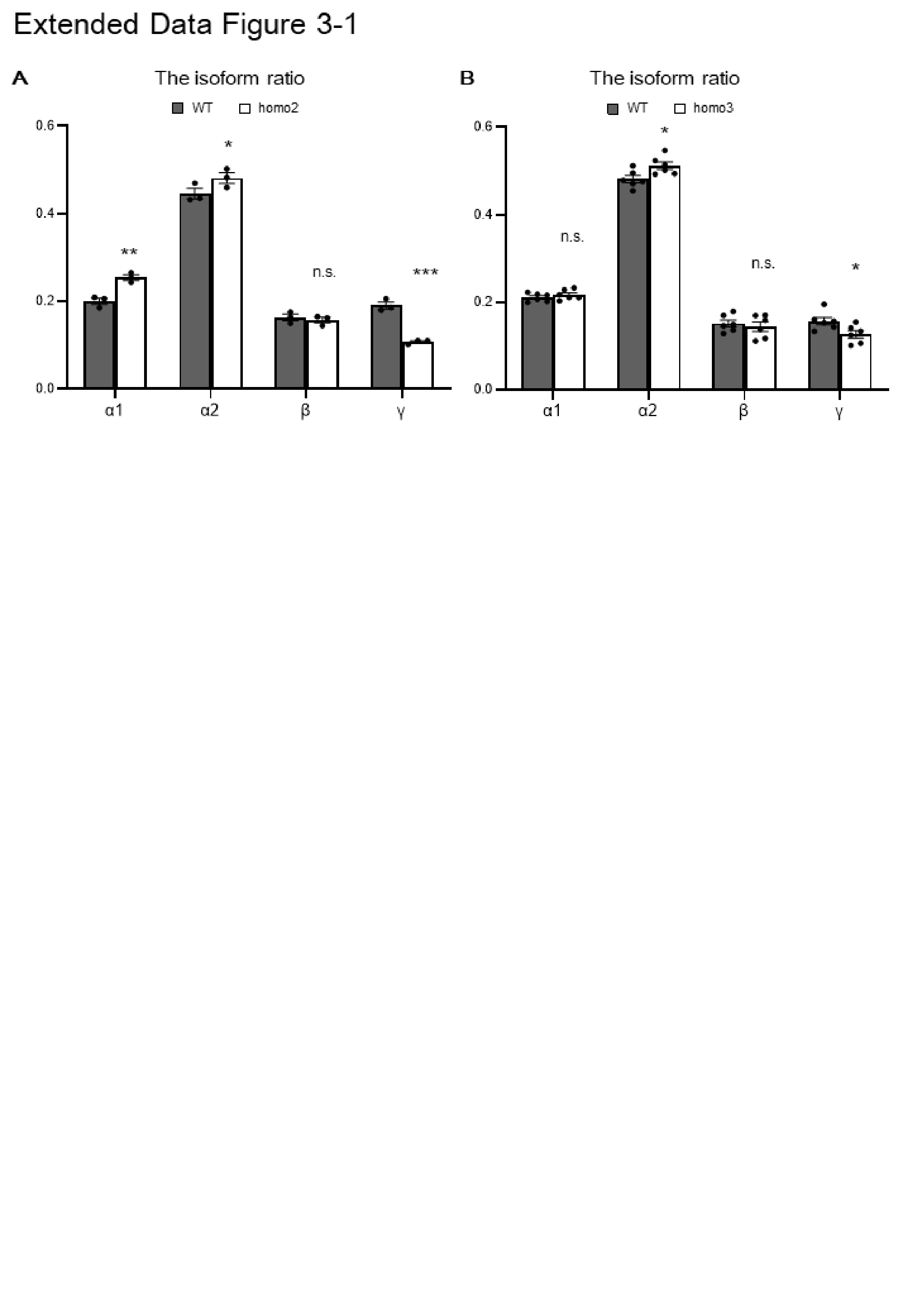

Supplement: Extended Data Figure 3-1 — The isoform changes in motor neurons with SYNGAP1 homozygous mutation. A, B, RNA from wild-type and homozygous 2 (homo2; A) or homozygous 3 (homo3; B) motor neurons were analyzed with RT-PCR, and the PCR products were analyzed with fragment analysis. Data are presented as the mean ± SEM n = 3 for homo2, n = 6 for homo3; *p < 0.05, **p < 0.01, ***p < 0.001; n.s., not significant, unpaired t test. Download Figure 3-1, TIF file. [file ns-JN-RM-0455-22-s03.tif]

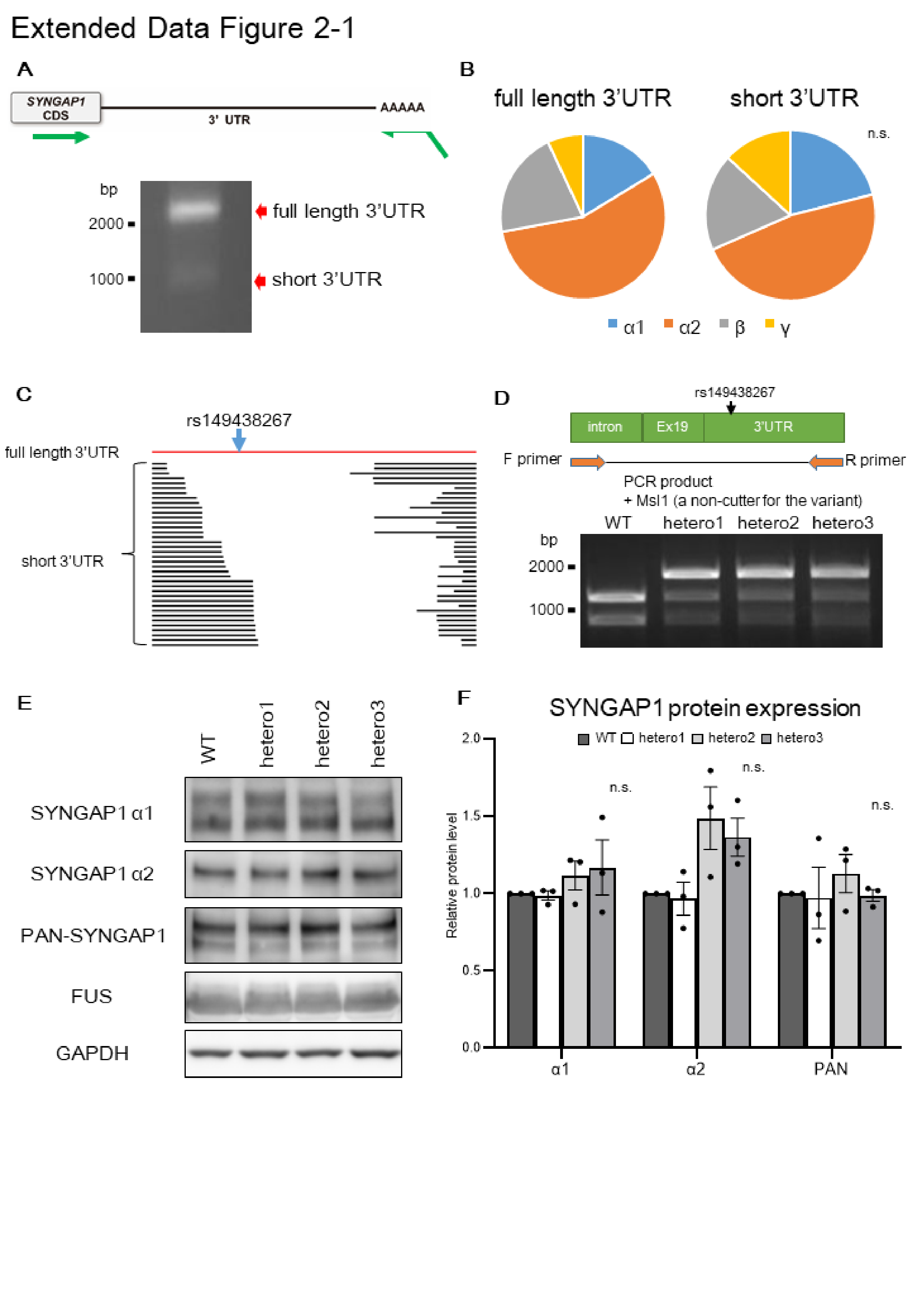

Supplement: Extended Data Figure 2-1 — SYNGAP1 3′UTR in iPSC-derived motor neurons. A, RNA from wild-type motor neurons was analyzed by 3′RACE and agarose gel electrophoresis. Note that short 3′UTR was present below the full-length 3′UTR. B, The bands in A were extracted and analyzed by Sanger sequencing to identify the ORF splicing patterns; full-length 3′UTR (n = 43); short 3′UTR (n = 38); n.s., not significant, χ2 test. C, The 3′UTR splicing patterns in B. The lengths of full-length 3′UTR (red line) and short 3′UTR (black lines). The spaces between the black lines represent the sites of 3′UTR skipping. D, RT-PCR was performed using the indicated primer sets to amplify DNA extracted from wild-type and edited heterozygous iPSCs. PCR products were digested by Msl1, a noncutter for the SYNGAP1 mutation, and analyzed with agarose gel electrophoresis. E, The lysates from wild-type and heterozygous iPSC-derived motor neurons were analyzed with Western blotting using the indicated antibodies. F, Quantification of the band intensities of the indicated proteins in E. n = 3; n.s., not significant, one-way ANOVA, Tukey's post hoc test. Download Figure 2-1, TIF file. [file ns-JN-RM-0455-22-s02.tif]

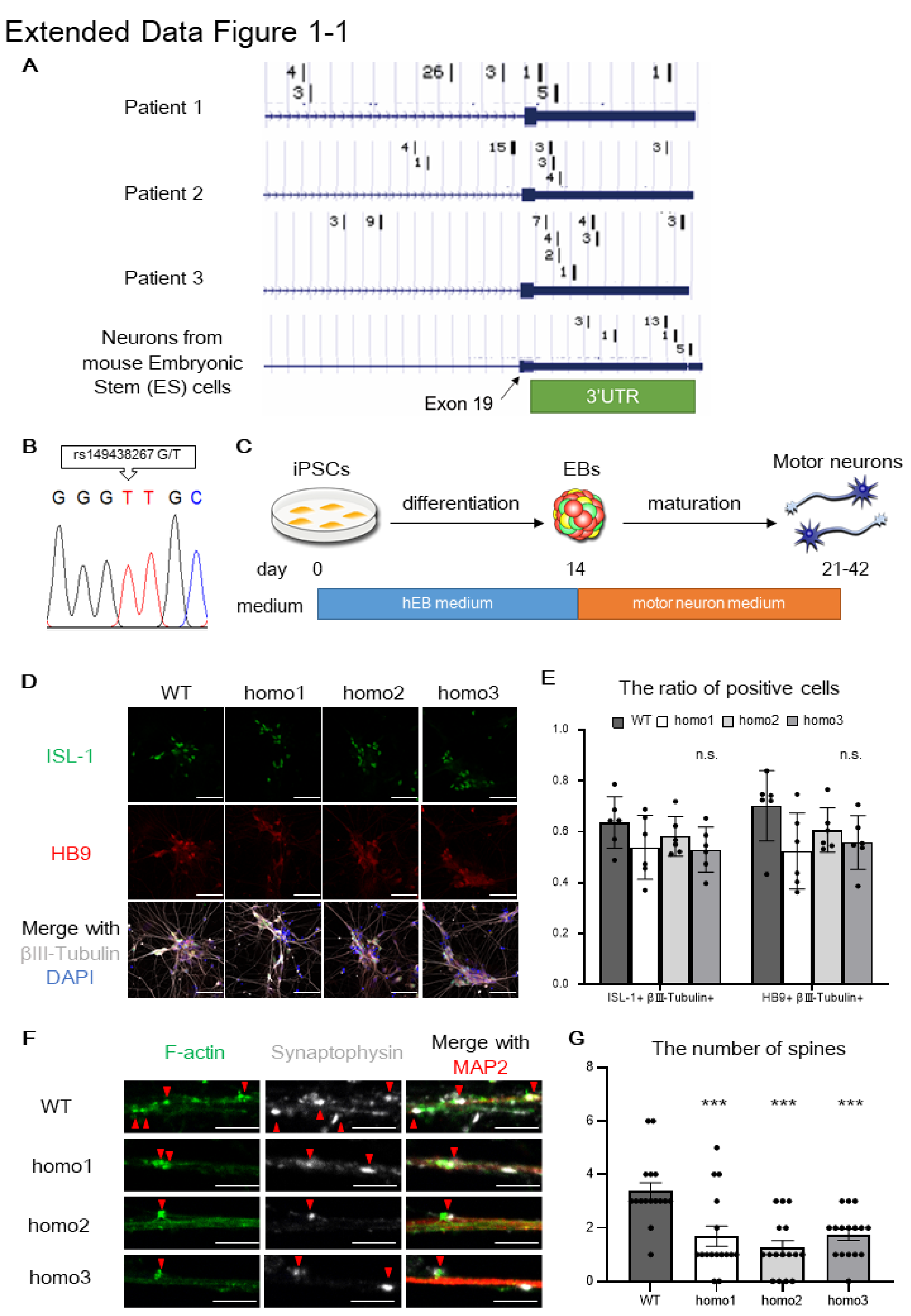

Supplement: Extended Data Figure 1-1 — The FUS binding sites at SYNGAP1 3′UTR and induced pluripotent stem cell (iPSC)-derived motor neurons. A, FUS CLIP-seq data from the temporal lobe of three epilepsy patients who underwent lobectomy (Nakaya et al., 2013). The number represents a pile-up of CLIP tags. B, The DNA sequence of iPSCs with the homozygous mutation (homo1). C, A schematic overview of the differentiation and maturation of iPSC-derived motor neurons from embryoid bodies (EBs). D, iPSC-derived motor neurons with the wild-type or homozygous mutation that were cultured for one week from EBs were immunostained for ISL-1 (green), HB9 (red), βIII-tubulin (white), and DAPI (blue). Scale bars: 10 µm. E, Quantification of the ratio of ISL-1/βIII-tubulin-positive and HB9/βIII-tubulin-positive cells per DAPI-positive cells. Data are presented as the mean ± SEM n = 6 fields from 3 independent wells; n.s., not significant, one-way ANOVA, Tukey's post hoc test (ISL-1), Kruskal–Wallis test, Bonferroni post hoc test (HB9). F, iPSC-derived motor neurons were immunostained for F-actin (green), MAP2 (red), and Synaptophysin (white). Scale bar: 5 µm. G, Quantification of the number of spines per 20 µm of dendrite length. Data are presented as the mean ± SEM n = 16 each from triplicate experiments; ***p < 0.001, Kruskal–Wallis test, Bonferroni post hoc test. Download Figure 1-1, TIF file. [file ns-JN-RM-0455-22-s01.tif]
